# Reconstructing the ecosystem context of a species: Honey-borne DNA reveals the roles of the honeybee

**DOI:** 10.1371/journal.pone.0268250

**Published:** 2022-07-13

**Authors:** Helena Kristiina Wirta, Mohammad Bahram, Kirsten Miller, Tomas Roslin, Eero Vesterinen

**Affiliations:** 1 Department of Agricultural Sciences, University of Helsinki, Helsinki, Finland; 2 Department of Ecology, Swedish University of Agricultural Sciences, Uppsala, Sweden; 3 Institute of Ecology and Earth Sciences, University of Tartu, Tartu, Estonia; 4 Department of Biology, University of Turku, Turku, Finland; University of Leipzig Faculty of Life Sciences: Universitat Leipzig Fakultat fur Lebenswissenschaften, GERMANY

## Abstract

To assess a species’ impact on its environment–and the environment’s impact upon a species–we need to pinpoint its links to surrounding taxa. The honeybee (*Apis mellifera*) provides a promising model system for such an exercise. While pollination is an important ecosystem service, recent studies suggest that honeybees can also provide disservices. Developing a comprehensive understanding of the full suite of services and disservices that honeybees provide is a key priority for such a ubiquitous species. In this perspective paper, we propose that the DNA contents of honey can be used to establish the honeybee’s functional niche, as reflected by ecosystem services and disservices. Drawing upon previously published genomic data, we analysed the DNA found within 43 honey samples from Northern Europe. Based on metagenomic analysis, we find that the taxonomic composition of DNA is dominated by a low pathogenicity bee virus with 40.2% of the reads, followed by bacteria (16.7%), plants (9.4%) and only 1.1% from fungi. In terms of ecological roles of taxa associated with the bees or taxa in their environment, bee gut microbes dominate the honey DNA, with plants as the second most abundant group. A range of pathogens associated with plants, bees and other animals occur frequently, but with lower relative read abundance, across the samples. The associations found here reflect a versatile the honeybee’s role in the North-European ecosystem. Feeding on nectar and pollen, the honeybee interacts with plants–in particular with cultivated crops. In doing so, the honeybee appears to disperse common pathogens of plants, pollinators and other animals, but also microbes potentially protective of these pathogens. Thus, honey-borne DNA helps us define the honeybee’s functional niche, offering directions to expound the benefits and drawbacks of the associations to the honeybee itself and its interacting organisms.

## Introduction

Species not only occur within ecosystems, they also function within them based upon their interactions. Thus, the niche of a species can be described from two angles, its Grinnellian niche which describes the environment in which the species lives [1, 2] and its Eltonian niche describing the impact the species has upon other taxa though interactions [3]. In order to comprehensively describe a species’ niche, these two perspectives should therefore be combined [4, 5]. The functional Eltonian niche remains notoriously difficult to establish, as it requires the establishment of all functions performed by the species in question. By contrast, the abiotic niche of a species is easier to determine, and as a consequence, the Grinnellian niche of species tends to be better understood for the majority of species.

To assess a species’ impact on its environment–and the environment’s impact on the species–we should pinpoint its links to surrounding taxa. The honeybee (*Apis mellifera*) provides a promising model system for such an exercise, as it is a functionally important species in ecosystems around the world. In fact, the honeybee is seen as the most important pollinator of global crops [6]. On top of their utility for pollination, honeybees are kept by humans for the production of honey. As a result of the human-bee association, there are over 90 million beehives in the world [7], each of which will host some 10 000–60 000 honeybees [8]. Thus, the honeybee is likely one of the most abundant insect species in the world, and globally affects a wide variety of ecosystems.

Nonetheless, the full suite of ecosystem functions provided by honeybees remain understudied. As ecosystem functions tend to be valued from a human-centered perspective, functional outcomes of ecosystem processes tend to be classified as ecosystem services or disservices [9, 10]. Ecosystem services are the benefits that people obtain from an ecosystem, whereas disservices are disadvantages and economic losses provided by the functions of organisms [10]. In this conceptual framework, the honeybee has been shown to sustain indisputable provisioning services in terms of honey production and regulatory services in terms of pollination [9–11]. Nonetheless, recent studies point to important disservices provided with the very same bee. Recently the honeybee has been shown to compete with wild pollinators as well as to spread pathogens of animals and plants [12–20].

As for any other organism, the quantification of honeybee impacts on its surroundings is laborious [e.g. 9–12]. This is a major challenge to modern ecology–as clearly, most ecologists will agree that the Eltonian [3] niche of a species captures something fundamentally important. Thus, we will use this paper to propose and illustrate how DNA-based methods may be used to provide new insight to the fundamental role of a species in the ecosystem in which it is immersed. For this purpose, we adopt the DNA stored in honey as our source of information.

**Fig 1:**
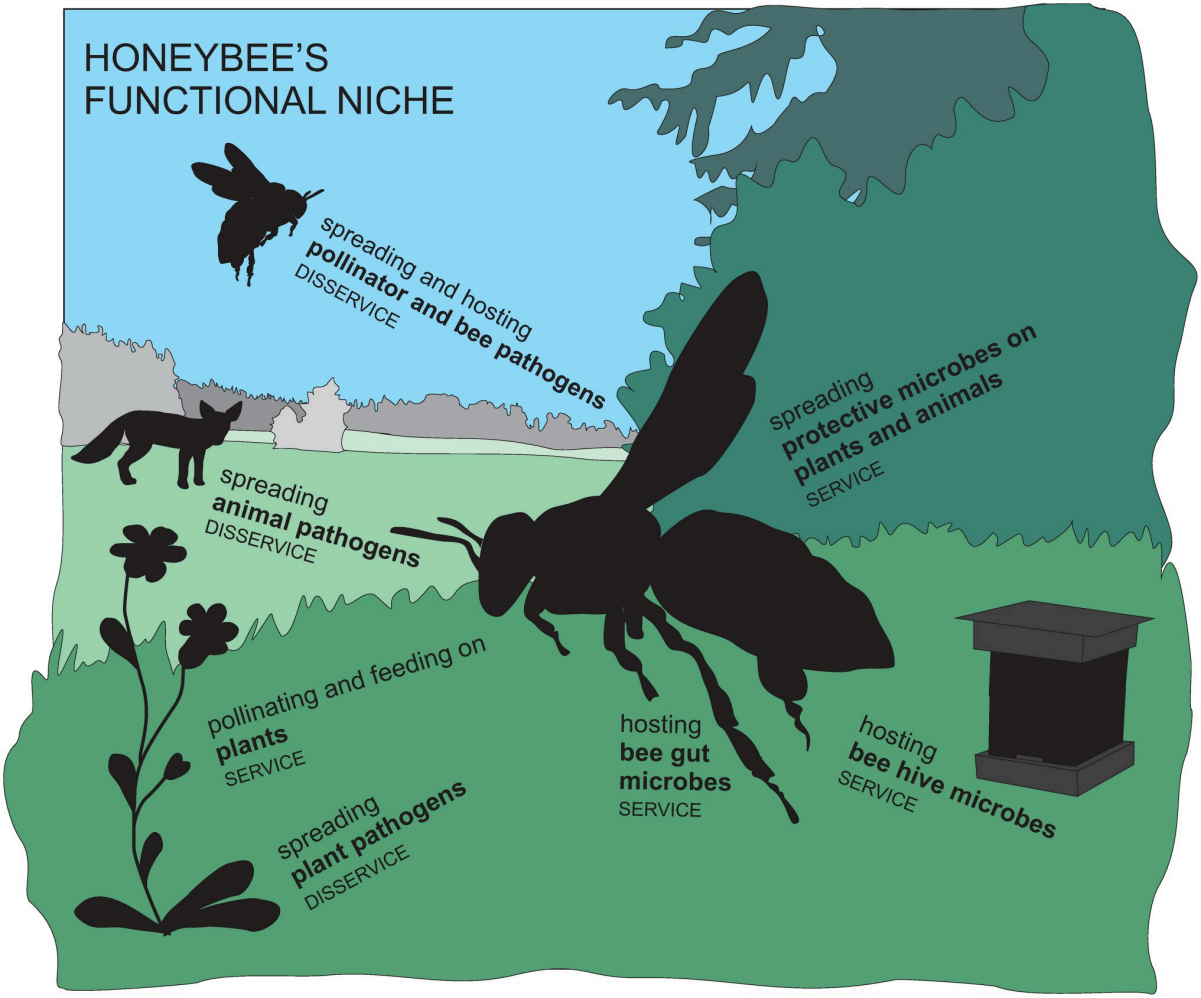
A conceptual figure of the functional niche of honeybees, and the ecosystem services and disservices the functions generate. A conceptual figure of the functional niche of honeybees drawing on previous research [14–17, 27, 28] and the taxa whose DNA we have identified from honey. The taxa as ecological groups are given in bold, with the likely function of honeybees related to this group above it. In small capitals is given whether the function could be considered an ecosystem service or disservice, either by the function honeybees do or what the taxa detected by DNA do to honeybees.

Using honey-borne DNA for the focal evidence comes with multiple benefits. DNA is well preserved in honey, and thus the DNA sequencing of honey reveals a wealth of taxa, from flowers to microbes [21–26]. These DNA traces are indicative of what organisms the honeybee has physically interacted with (either intentionally or unintentionally). Therefore, our aim with this perspective paper is to use honey-borne DNA to show the potential of DNA-based methods in establishing a species Eltonian niche, and to tentatively suggest how it translates to the provisioning of ecosystem services and disservices (Fig 1).

Despite different levels of detectability of different interactions and interaction types [29, 30], we argue that the links uncovered by DNA-based identification provides a new, increasingly quantitative view of the honeybees’ position in an ecosystem. We support this claim by measuring the interactions of honeybees from two different perspectives: their incidence as interaction partners and the relative importance of this interaction when it occurs.

The data used for our study derive from a previous paper [23], in which we ask whether the taxonomic contents of honey might be used to identify the country of origin [23]. In the current study, we approach the same data from a novel perspective, asking what the taxonomic and functional groups detected in honey will reveal about the honeybee’s role in its environment.

## Materials and methods

### Honey samples

To describe the role of honeybees in their environment, we reutilize metagenomics and DNA metabarcoding data from a recently published study [23] for 46 honey samples from North Europe. The initial dataset comprises 19 honey samples from Finland, 19 from Sweden and eight from Estonia; however, three samples (one from Estonia, one from where and one from where) yielded low sequence numbers from metagenomics sequencing, and were therefore omitted from metagenomics analysis (for origins of samples, see map as S1 Fig). Eight Finnish and nine Swedish samples were provided by beekeepers directly (seven and eight, respectively, provided data for metagenomics), while most of the samples (eleven from Finland, ten from Sweden and all eight samples from Estonia) were obtained from the retail market (S1 Fig). The latter type of samples is likely to consist of honey combined from more than one beekeeper [23]. To evaluate whether the two types of samples differed in terms of the key metrics examined, we performed a separate analysis–finding commensurate metrics (S1 Text and S2 Fig). Thus, the two sample types are henceforth used interchangeably throughout this study.

Our overall material provides a sample of honeybee colonies across the three target countries. They are therefore jointly representative of the honey and honey contents for this region, thereby providing an opportunity to identify the ecological associations and functions of the honeybee in Northern Europe. The biota among these three neighbouring countries is very similar, and earlier honey analyses, based on morphological identification of pollen [31–33], have shown the honey of these three countries mainly originate from the same plants. As a result, the bees can be assumed to sample from what is essentially the same regional pool of potential associates. Thus, we may use the mean relative read abundance across samples, and the observed frequency of occurrence of a taxon across the full set of honey samples, as direct metrics of association across this region (see section *Relative read abundance and frequency of occurrence*).

### DNA extraction, metagenomics and metabarcoding methods

We use the data from [23], based on the exact same methods. We provide a short overview of the main methods applied here in the main text, and offer full details in Supplementary Material (S2 Text). For each honey sample, two DNA extractions were conducted, each using 20 g of honey to assure a sufficient amount of DNA for the analyses. One DNA sample was prepared to be used for metagenomics and one for metabarcoding [23]. For each DNA sample the 20 g of honey was divided into two, to fit 10 g of honey and 30 ml of water into a 50 ml tube. Then the two subsamples, each consisting of 10 g of honey, were diluted to 30 ml of DNA-free water. After centrifugation, the pellets of two subsamples were combined back into one DNA sample, and the total DNA was extracted with DNeasy Plant Mini Kit (Qiagen, Germany).

For metagenomics, the DNA was fragmented into 150 base pair pieces prior the library preparation and sequencing with Illumina NextSeq 500 Sequencer [23]. Both the sequencing and bioinformatic processing of reads were done by University of Helsinki’s Biomedicum Functional Genomics Unit [34]. After quality filtering, taxonomic labels were assigned to the sequencing reads from a custom-build National Center for Biotechnology Information [35] non-redundant nucleotide database, as accessed in September 2019.

The DNA sample for metabarcoding was used to identify bacterial, fungal and plant taxa in the samples. The bacterial and fungal gene regions are the same as in worldwide research campaigns [36, 37], with the same relatively universal primer pairs; 16S for bacteria, with two primer set referred to as 16Sa and 16Sb, and ITS2 for fungi. The same approach of commonness, universality, and practicality was used to choose the primers targeting three gene regions (ITS2, rbcLa and trnL) to examine the plant DNA contents in honey samples [23]. All the gene regions were amplified twice, after which they were indexed with unique indexes and sequenced on an Illumina MiSeq sequencer. For the sequence reads the paired end reads were merged, primers removed, reads quality filtered, dereplicated and clustered to OTUs, which were then assigned taxonomically to a specific reference database for each gene region. Reference databases were accessed in April 2019. The DNA sequence datasets are available in the Sequence Read Archive repository, in the BioProject PRJNA662672 (https://www.ncbi.nlm.nih.gov/sra/PRJNA662672).

We note that the read abundance observed by metagenomics are likely to be more closely reflective of the original DNA contents of the sample, since no amplification step is required before sequencing. Thus, we focus our results on the metagenomics analysis. We then compare our findings from metagenomics with those from metabarcoding, to show how each method performs in uncovering honey-borne biodiversity.

### Relative read abundance and frequency of occurrence

To establish what taxa North-European honeybees interact with or encounter, and with what approximate frequencies, we used relative read abundance and frequency of occurrence across samples. As a crude measure of the relative strength of interaction, we calculate the mean relative read abundance (mean RRA), i.e. the proportion of sequence reads assigned to a taxon out of all sequence reads of a sample, across samples [38]. As a measure of the incidence of the interaction, we calculate the frequency of occurrence (%FOO), i.e. the proportion of samples in which a taxon occurs across samples [38]. The latter metrics were calculated for all taxa using metagenomics data and for bacterial, fungal and plant genera based on metabarcoding data. We note that these metrics do not allow for a direct evaluation of a functions importance, but they can be used to describe the different functions’ commonness overall.

For assessing the diversity of taxa in honey as well as the ecological roles associated with them, we focus on the taxonomic level of the genus. This solution was based on the genus being the lowest taxonomic level to which the majority of reads could be assigned (see [23]), and on taxonomic assignments for 16S metabarcoding of bacteria being restricted to genera [39]. For metagenomics data, we also name the species with highest mean RRA, but since only a smaller portion of all reads could be been assigned to species, we refrain from further analyses at the species level.

### Functions of microbes

To establish the functional roles of microbes detected by DNA in honey, and the functions of honeybees related to them, we classify the most abundant cellular microbial genera based on their role for the honeybee itself or their general role in the environment based on literature. For microbes closely related to bees, we classify them into bee gut microbes (as occurring commonly in the honeybee’s gut); beehive microbes (as being part of the microbe community known from the hives); and bee pathogens (as including microbes known to be pathogenic for other pollinators as well). For microbes without a direct association to the bee itself, we classify them as plant pathogens; animal pathogens; and microbes known to beneficial or neutral from the perspective of plants and animals. Since many microbe genera could have multiple roles, they could in principle fit into more than one of these groups. Nonetheless, we have here chosen to classify them from the honeybee’s point of view, thus primarily assigning a genus into either the bee gut, bee hive or bee pathogen group. We only consider genera with ≥0.01% mean RRA based on metagenomics for this classification. We quantify the groups by summing up the mean RRA of the genera and by averaging across the %FOO of the genera in each group. For comparison, we show also plants’ mean RRA and %FOO based on metagenomics.

To examine the functional roles of the microbes detected based on DNA metabarcoding, the functional groups of each OTU was determined based on FAPROTAX [40] for bacteria, as using data on both 16Sa and 16Sba primer pairs. The functional guilds of the fungi from metabarcoding were identified according to FungalTraits and FUNGuild [41, 42]. For fungi, we consider the placement of the genus level identification to a functionality guild only with either probable or highly probable confidence ranking provided in the database [42].

## Results

### Viral and bacterial DNA is more common in honey than is plant DNA

**Fig 2:**
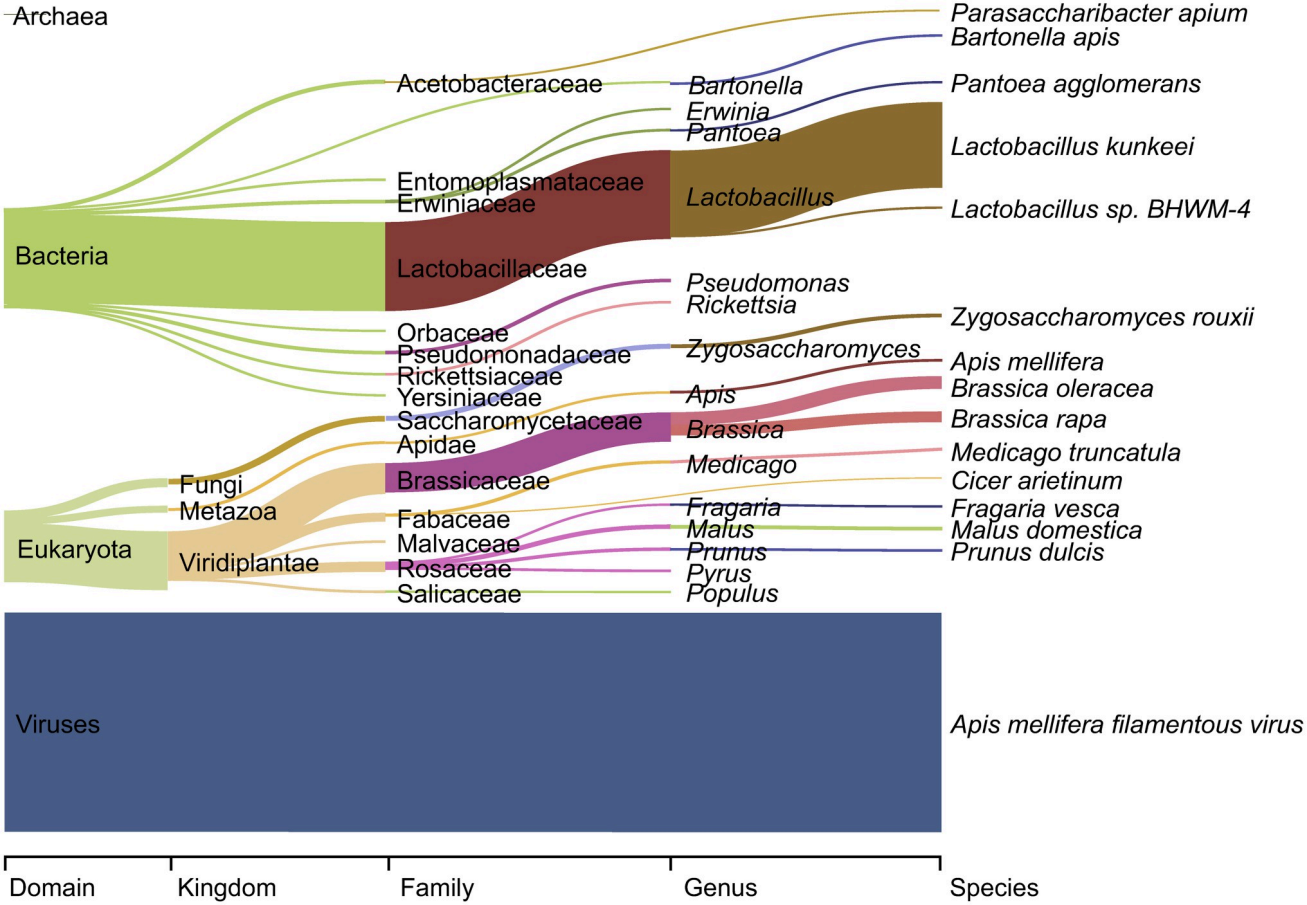
Sankey diagram of the most abundant taxa detected by DNA metagenomics in North-European honey samples. The width of the bar is proportional to the mean RRA of the taxa, with the fifteen taxa with the highest mean RRA shown at each taxonomic level. The graph was created with Pavian [43].

To establish the role of the honeybee in North European ecosystems, the occurrence and abundance of associated taxa in the DNA contents of honey need to be identified and quantified. Based on metagenomics, the reads found in North-European honey samples originate mainly from viruses (mean RRA 40.2% (SD ±30.0)), as derived from a single species, the *Apis mellifera* filamentous virus (40.2%, ± 30.0%; Fig 2). A smaller fraction of the total read numbers were of bacterial (16.7% ± 18.4) or eukaryotic origin (13.3% ± 8.5) and only a minor fraction (0.02% ±0.02) belong to Archaea (Fig 2). Yet, a large percentage (mean RRA 29.8% (SD ± 14.2)) of the reads could not be identified.

Within eukaryotes, reads assigned to plants (Viridiplantae) on average comprised 9.4% of total reads (± 7.3), while those originating from fungi and metazoa only comprised 1.1% (± 3.1) and 2.4% (± 1.3), respectively.

### Relatively abundant microbes are taxonomically diverse

A large number of cellular microbial genera were found to be relatively abundant in the metagenomics data. The bacterial genus with the highest relative read abundance is *Lactobacillus* with mean RRA of 13.7 (±17.5) and the second most abundant *Pseudomonas* with 0.3 (±0.5). Most of the genera among the ones with the highest relative read abundances were also detected in all or nearly all samples, with the exceptions of *Mesoplasma*, *Entomoplasma* and *Melissococcus*, which all occurred at lower incidence (%FOO 74.4, 65.1 and 55.8 respectively; Fig 2, Table 1 and S1 Table). Among fungi, *Zygosaccharomyces* was the most abundant genus with a mean RRA of 0.65 (±2.54). The second most abundant fungus was *Claviceps* with a mean RRA of 0.05 (±0.32), although it occurred in only 18.6% of the samples (Table 1 and S2 Table). At the species level, the relatively most abundant taxon was the lactic acid bacterium *Lactobacillus kunkeei* (13.2%, ±17.1) and the second relatively most abundant was the yeast *Zygosaccharomyces rouxii* (0.5%, ±1.9; Fig 2).

**Table 1:** The relatively most abundant microbial genera with their ecological group based on metagenomics.

group	genus	RRA		%FOO	ecological group	example species	references
		mean	SD				
bacteria	*Lactobacillus*	13.71	17.52	97.67	bee gut	*L*. *kunkeei*, *L*. *Firm-4*, *L*. *apis*	[44–47]
fungi	*Zygosaccharomyces*	0.65	2.54	95.35	bee hive	*Z*. *mellis*, *Z*. *rouxii*	[48]
bacteria	*Pseudomonas*	0.32	0.48	93.02	plant protective	*P*. *fluorescens*, *P*. *viridiflava*	[49]
bacteria	*Parasaccharibacter*	0.25	0.32	97.67	bee hive	*P*. *apium*	[50, 51]
bacteria	*Pantoea*	0.19	0.32	97.67	plant protective	*P*. *agglomerans*	[52]
bacteria	*Rickettsia*	0.17	0.70	93.02	animal pathogen	*R*. *felis*, *R*. *asiatica*	[53]
bacteria	*Erwinia*	0.12	0.14	97.67	plant pathogen	*E*. *amylovora*, *E*. *tasmaniensis*	[49, 54]
bacteria	*Acinetobacter*	0.10	0.24	86.05	bee gut	*A*. *haemolyticus*, *A*. *apis*	[55]
bacteria	*Mesoplasma*	0.09	0.30	74.42	neutral or protective on plants and insects	*M*. *florum*	[56, 57]
bacteria	*Bartonella*	0.07	0.29	90.70	bee gut	*B*. *apis*	[44, 45]
bacteria	*Serratia*	0.07	0.15	95.35	bee pathogen	*S*. *marcescens*, *S*. *symbiotica*	[27]
bacteria	*Entomoplasma*	0.06	0.19	65.12	neutral or protective on plants and insects	*E*. *melaleucae*, *E*. *lucivorax*	[57]
bacteria	*Arsenophonus*	0.06	0.11	88.37	bee pathogen	*A*. *nasoniae*	[58, 59]
bacteria	*Spiroplasma*	0.06	0.08	95.35	bee pathogen	*S*. *apis*, *S*. *melliferum*	[27]
bacteria	*Streptomyces*	0.05	0.03	100.00	neutral or protective on plants and insects	*S*. *hygroscopicus*, *S*. *cattleya*	[60]
fungi	*Claviceps*	0.05	0.32	18.60	plant pathogen	*C*. *purpurea*	[61]
bacteria	*Gilliamella*	0.05	0.17	83.72	bee gut	*G*. *apicola*	[44]
bacteria	*Rahnella*	0.05	0.14	97.67	bee hive	*R*. *aquatilis*	[62, 63]
bacteria	*Bacillus*	0.04	0.03	100.00	bee gut	*B*. *weihaiensis*, *B*. *cereus*	[64, 65]
bacteria	*Lactococcus*	0.03	0.06	93.02	bee gut	*L*. *lactis*, *L*. *garvi*	[44]
bacteria	*Snodgrassella*	0.03	0.06	88.37	bee gut	*S*. *alvi*	[44]
bacteria	*Melissococcus*	0.03	0.07	55.81	bee pathogen	*M*. *plutonius*	[27]
bacteria	*Frischella*	0.02	0.06	83.72	bee gut	*F*. *perrara*	[44]
fungi	*Saccharomyces*	0.02	0.07	100.00	bee hive	*S*. *paradoxus*, *S*. *jurei*	[66]
bacteria	*Streptococcus*	0.02	0.02	90.70	animal pathogen	*S*. *mitis*, *S*. *suis*, *S*. *pneumoniae*	[67]
bacteria	*Enterobacter*	0.02	0.03	100.00	bee hive	*E*. *cloacae*, *E*. *asburie*	[68]
bacteria	*Commensalibacter*	0.02	0.02	97.67	bee gut	sp	[69]
bacteria	*Clostridium*	0.02	0.02	93.02	animal pathogen	*C*. *botulinum*, *C*. *perfringens*	[70]
fungi	*Aspergillus*	0.02	0.02	93.02	bee pathogen	*A*. *novofumigatus*, *A*. *nomius*	[28]
bacteria	*Lonsdalea*	0.02	0.07	25.58	plant pathogen	*L*. *britannica*	[71]
bacteria	*Staphylococcus*	0.02	0.02	72.09	animal pathogen	*S*. *aureus*, *S*. *equorum*	[72]
bacteria	*Paenibacillus*	0.01	0.01	100.00	bee pathogen	*P*. *larvae*	[27]
bacteria	*Yersinia*	0.01	0.03	86.05	animal pathogen	*Y*. *enterocologica*, *Y*. *ruckeri*	[73]
bacteria	*Enterococcus*	0.01	0.02	88.37	bee gut	*E*. *faecalis*, *E*. *silesiacus*	[47]
bacteria	*Vibrio*	0.01	0.02	90.70	animal pathogen	*V*. *vulnificus*	[74]
bacteria	*Klebsiella*	0.01	0.02	90.70	bee hive	*K*. *pneumoniae*, *K*. *aerogenes*	[44, 75]
bacteria	*Leuconostoc*	0.01	0.02	90.70	bee hive	*L*. *mesenteroides*	[58]
bacteria	*Burkholderia*	0.01	0.01	100.00	bee hive	*B*. *cepacia*	[76, 77]
bacteria	*Mycoplasma*	0.01	0.02	93.02	animal pathogen	*M*. *gallisepticum*, *M*. *yeatsii*	[78]
bacteria	*Bifidobacterium*	0.01	0.02	86.05	bee gut	*B*. *asteroides*, *B*. *dentium*	[44]
bacteria	*Proteus*	0.01	0.03	67.44	animal pathogen	*P*. *hauseri*, *P*. *mirabilis*	[79]
bacteria	*Escherichia*	0.01	0.01	83.72	animal pathogen	*E*. *coli*, *E*. *albertii*	[80]
fungi	*Naumovozyma*	0.01	0.04	74.42	bee hive	*N*. *dairenensis*, *N*. *castellii*	[81]
fungi	*Cryptococcus*	0.01	0.01	100.00	animal pathogen	*C*. *neuformans*, *C*. *gattii*	[82]

The relatively most abundant cellular microbial genera as quantified by metagenomics. For each taxon, we show its ecological group based on literature (for classification, see section Materials and methods: *Functions of microbes*). Included in the table are taxa with a mean RRA ≥0.01% in the metagenomics data. For each genus we provide examples of species identified from the North-European honey samples.

There was large variation in how well the bacterial and fungal genera found relatively most abundant by metagenomics were also detected by metabarcoding. For both bacteria and fungi, most of the genera were detected by metabarcoding (S2 and S3 Tables), but some genera found relatively abundantly by metagenomics were not detected by metabarcoding. This is likely due to selective amplification, despite the primers used in this study being commonly used in universal campaigns aimed at characterizing bacterial [36] and fungal communities [37, 41], and to lack of sufficient variation between closely-related taxa in these gene regions, delimiting the assignment of reads to specific genera.

### Microbes hosted by honeybees dominate abundances in bee guts and hives

**Fig 3:**
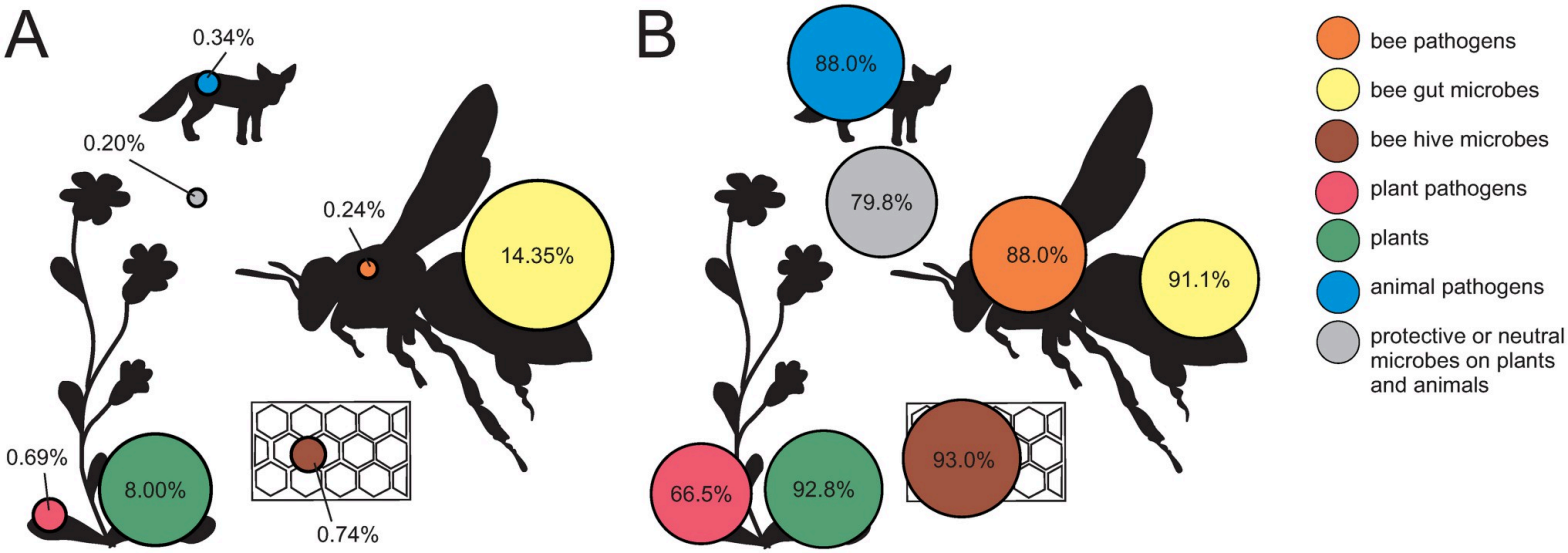
The abundance and frequency of ecological groups of microbes and plants based on DNA in honey. Shown are microbe genera detected with a frequency of at least 0.01% of mean RRA across taxa based on metagenomics, as grouped based on their role for honeybees and in the environment (Table 1). Also shown are values for plant genera detected with a mean RRA of at least 0.01%. In panel A, we show the average RRA of the genera summed across taxa within each group. In panel B we show the average FOO% across these genera. The size of the circles are scaled according to the value of RRA and FOO%, respectively.

To understand the functions provided by the honeybee, we need to characterize the different roles of the microbes which they associate with. For the microbes commonly found in the guts and the hives of the honeybee, the bee will mainly serve as a host and the microbes appear to be mainly beneficial to this host. The ecological group of bee gut microbes was the relatively most abundant one detected by DNA in honey based on metagenomics, considering cellular organisms (Fig 3, Table 1). The relative abundance of this group is mostly attributable to the many reads assigned to the genus *Lactobacillus*, and to the species *L*. *kunkeei* especially. Microbes such as *Zygosaccharomyces*, *Parasaccharibacter* and *Rahnella*—commonly found in the bee hives—were the second most common microbial group, followed by groups of bee pathogens, animal pathogens and plant pathogens, each showing similar levels of mean RRA as the beehive microbes. Due to their potentially close associations, pathogens are likely spreaded by honeybees to other honeybee colonies as well as to other pollinators. This vector function of honeybees applies to the other groups of plant and animal pathogens, too, but also to the microbes beneficial to plants. From the bee’s perspective, the latter spread is unintentional and will likely have no effect on the bee itself.

As another way to measure functioning of the taxa, we may use the bacterial and fungal sequences obtained by metabarcoding to classify them to functional groups (OTUs for bacteria, S5 Table, and genera for fungi, S6 Table). The most common bacterial function based on mean RRA was fermentation (mean RRA 64.76%, ±32.28). This likely refers to the highly abundant *Lactobacillus kunkeei*, which is a fructophilic lactic acid bacterium [83], as well as to other lactic acid bacteria living in the gut of the honeybee. These bacteria are also commonly found on flowers [44, 46, 47]. Animal parasites and symbionts, as well as microbes involved in aromatic compound degradation and nitrogen fixation are the next most abundant groups (9.47% ±9.06, 6.87% ±7.88, 3.10% ±6.52, respectively), followed by human pathogens based on the primer pair 16Sa (3.04%, ±6.08). Fermentation, intracellular parasites and nitrogen fixation were the most abundant functions based on 16Sb (37.15 ±33.56, 25.34% ±26.65, 11.33% ±17.77, respectively, S5 Table). Plant pathogenic bacteria were found in two thirds (60.1%) of the samples, although their mean RRAs are not among the highest ones for these functional classes (S5 Table). Instead, for fungi plant pathogens are the most abundant group with 44.7% mean RRA (±27.1) and have an occurrence of 100% (S6 Table). Animal pathogens were also highly abundant. For the fungi, many groups are likely associated with several different functions (S6 Table).

### Crop plants dominate as the food providers and pollination partners of the honeybee

**Fig 4:**
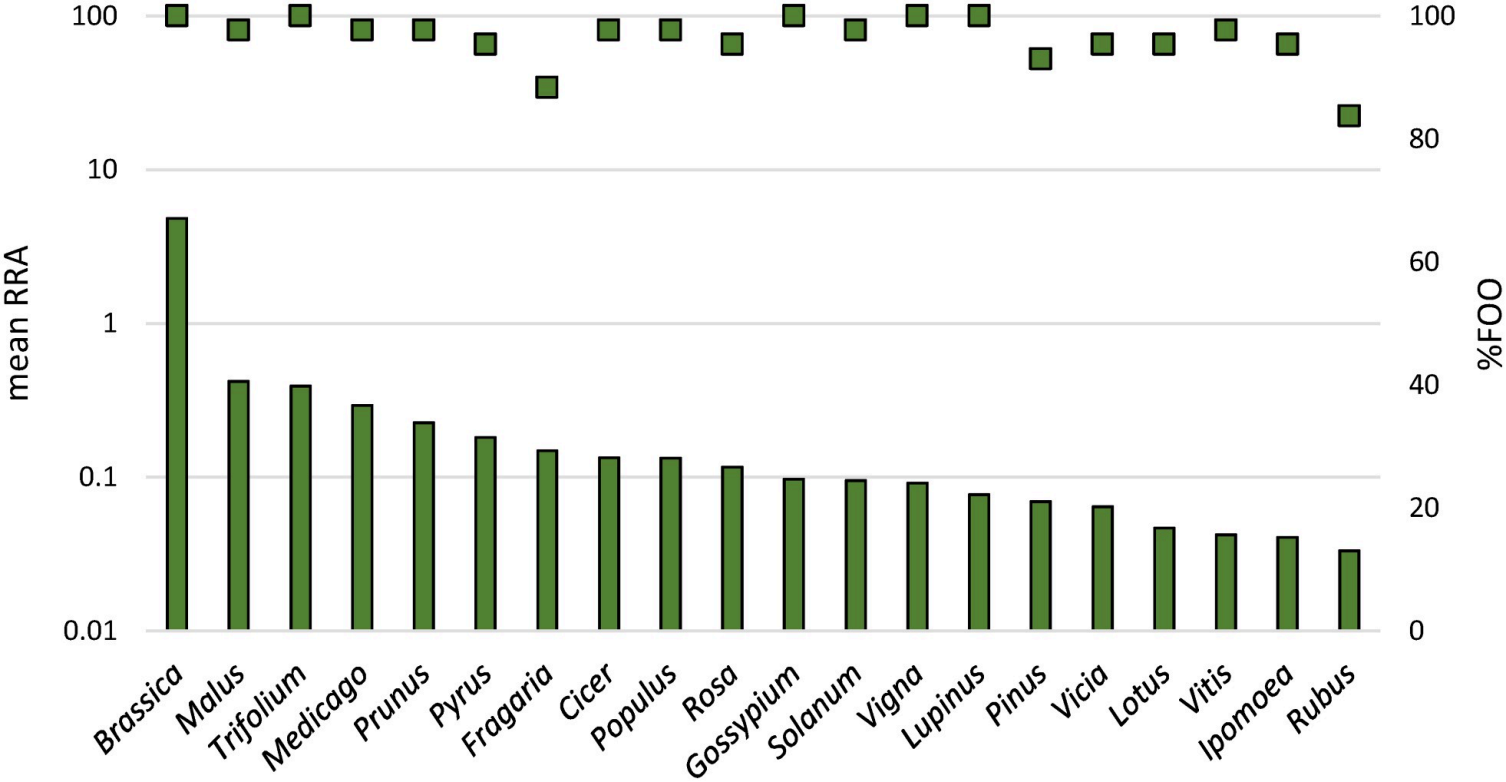
The relatively most abundant and frequent plant genera based on the DNA in honey samples. The twenty plant genera with the highest mean RRA based on metagenomics (y-axis on the left), shown with %FOO (y-axis on the right). Note the logarithmic scale used for RRA.

Of the plants providing nutrients–nectar and pollen–to honeybees, *Brassica* stood out as the single genus being far more abundant than other genera. This genus accounted for a mean RRA of 4.8% (± 5.3), followed by the genera *Malus* (0.42%, ±0.68) and *Trifolium* (0.39%, ±0.47; Figs 2 and 4, S4 Table). Among the plant genera that were among the most relatively abundant ones, there was a lot of variation in their frequency of occurrence from 48.8% (*Rubus*) to 100% (*Gossypium*; Fig 4, S4 Table). At the species level, the relatively most abundant plant taxa identified, based on their mean RRA, were *Brassica oleracea* (1.93%, ±2.53), *B*. *rapa* (1.72%, ±1.82), *Trifolium repens* (0.36%, ±0.44), *Malus domestica* (0.33%, ±0.47) and *Medicago truncatula* (0.29%, ±0.33).

## Discussion

In this paper we demonstrate an approach for characterizing the Eltonian niche of a species. Using the honeybee as an example, we suggest that DNA hidden in honey gives tangible insights into the interactions of honeybees, by revealing the multitude of taxa that honeybees have been in contact with. For another species, DNA in another type of substance the species is tightly connected with, could serve as a similar sample of functions. While the DNA in honey samples can originate from both living and dead organisms, the consistent occurrence of the DNA of a given organism at substantial read counts could be taken as a sign of a frequent or strong interaction or contact between the bee and this organism. By characterizing the taxa honeybees associate with across a large set of samples, and by defining the ecological role of each taxon, we gain a novel perspective on the bee’s role in the ecosystem, and the ecosystem functions it is likely involved in. Based on these findings, we propose that DNA-based approaches can provide a novel and versatile tool to unlocking the Eltonian niche of other species than the bee as well.

### Plants form the main interaction partners of bees, and the main platform on which other functions play out

As honey is made of nectar, collected by the honeybees from flowers, it would be logical to assume that most of the tissue–and DNA–found in honey would originate from plants. Yet, in our samples, reads originating from viruses and bacteria are far more common than reads from eukaryotes altogether. Nevertheless, most of eukaryotic reads belong to plants. The main proportions of reads in honey belonging to viral and bacterial origin are supported by three other recent metagenomics studies on honey [21, 22, 24]. While the previous studies had only a few samples (two, three and four per study) from Italy and Greece [21, 22, 24], our results together with the results from these South European honey samples confirm the dominance of microbial DNAs in honey to be widespread and generic. This is suggestive of a variety of functions which honeybees may supply, as discussed below.

While flowering plants are the most important resources for honeybees feeding on nectar and on pollen, they are also the likely platforms on which most or all of the ecosystem services and disservices provided by honeybees will take place. While the–in itself unintentional–transfer of pollen from one flower to another renders honeybees as pollinators, these same movements and visits make honeybees vectors of microbes as well. Microbes occur on pollen, in nectar, as well as on petals of flowers [84–86], and all of these can be dispersed by pollinators [87]. Therefore, it is of interest to examine which plant species are the targets of honeybees functions most abundantly and frequently, as these would be the flowers which honeybees pollinate and among which they transfer microbes the most.

The most abundant plant genus in North-European honeys, both based on metagenomics and metabarcoding, is *Brassica*, with *B*. *oleraceae* and *B*. *rapa*. Among these, different types of rape, oilseed rape and turnip rape are widely cultivated in all the three countries from which the honey samples originate [88, 89]. Our data would suggests that the honeybee uses *Brassica* frequently and abundantly, and *Brassica* is thus a large contributor to honey crops. The same applies to clover, *Trifolium* [32, 33, 90]. The genera *Malus*, *Prunus* and *Fragaria* were likewise commonly and abundantly found in the DNA contents of honey, matching previous records from North-European honeys as based on morphological identification of pollen (melissopalynology) [32, 33]. Yet, the high relative abundance and frequent occurrence of *Medicago*, *Populus* and *Solanum*, all of these common both by metagenomics and metabarcoding, has not been previously documented in North-European honey through melissopalynology [32, 33, 90]. It thus appears that DNA-based identification of plants now brings new resolution to detecting links between bees and plants.

The core functions defining the honeybee’s niche are thus in the feeding on the above listed plants, and in the simultaneous pollination of them. This reflects the major ecosystem services provided by honeybees in Northern Europe, as resulting from the pollination of cultivated plants such as different types of rapes, clovers, apples, plums and strawberries.

In regard to microbes affecting plants, the honeybees in Northern Europe are likely to facilitate the dispersal of the plant pathogens of the genus *Erwinia* [49, 54] the most. The fire blight pathogen *E*. *amylovora* is known to be spread by honeybees to plants [49]. Yet, in the honey samples, the genera *Pseudomonas* and *Pantoea* are relatively more abundant than plant pathogens. The two latter genera include species which act as biocontrol agents against *E*. *amylovora* and are commonly found in bee hives [49, 52]. Also, the genera *Mesoplasma*, *Entomoplasma* and *Streptomyces*, among the relatively abundant taxa in these honey samples, have an either protective or neutral effect on plants and insects on which they occur.

### Disservices emanating from the honeybee

For the pathogenic microbes detected, the pathogens affecting honeybees themselves are likely to be dispersed among honeybee colonies as well as among other pollinators. Of special interest is the dominant Apis mellifera filamentous DNA-virus (AmFV) with on average 40.2% (±30.0) of total reads per sample, of all DNA detected. This finding is in line with the South European honey samples [21, 22, 24] suggesting the AmFV is commonly the dominant organism in honey samples in Europe. AmFV is a large double-stranded DNA virus of honeybees found in many different tissues [91]. Its full genome has been sequenced [92] making it easily detectable by metagenomics. It is only weakly pathogenic and found very commonly in honeybees throughout the world [93, 94], as well as in other bee species [95]. The virus has been suggested to spread among the bees by food exchange [94], offering a straightforward path into honey. The prevelance of this virus suggests that it could be readily dispersed to other colonies and to other pollinators. Yet, without knowing if its impact is negative, it should not be labelled as a disservice.

With regard to cellular microbes, the genus *Serratia*, with *S*. *marcescens* causing septicemia [27] is the relatively most abundant bee pathogen genus and it occurs in nearly all samples. Apart from honey samples, it is frequently found in honeybees, not always being infective to honeybees, yet it is an opportunistic pathogen also on other insects and plants [96]. *Spiroplasma* species cause May disease [27] and *Arsenophonus nasoniae* is associated with poor hive health and colony collapse disorder [58, 59]. *Aspergillus* species cause stone brood disease, *Paenibacillus larvae* causes American Foulbrood and *Melissococcus plutonius* European Foulbrood [27, 97] of which the Foulbroods are the most devastating bee diseases causing large economic losses worldwide [27, 98, 99]. While these genera have been mainly studied as for their pathogenic effects to honeybees, many of these pathogens have been found in wild pollinators in different parts of the world [12, 27, 100, 101]. The dispersal of pathogenic microbes is likely to occur in both directions, from managed honeybees to wild pollinators and *vice versa* [12, 102]. Besides that the relative abundance and frequency of occurrence of these bacterial genera may reflect the wellbeing of the bee colonies in concern, they also show which pathogens honeybees are most likely dispersing among different pollinators.

Many of the pathogens detected in the DNA content of honey infect mammals or vertebrates in general. Genus *Rickettsia*, species of which cause serious diseases to mammals and are transmitted by arthropods [53], was here detected as the relatively most abundant animal pathogen. Also species of *Streptococcus*, *Clostridium* and *Staphylococcus* may be pathogenic to mammals, including humans [67, 70, 72, 103]. Some of these pathogenic microbes are known to enter honey from the environment, such as *Clostridium botulinum* [70, 103]. Such microbes may be dispersed by the honeybee, counting as an ecosystem disservice. Yet, the DNA of genera pathogenic to humans may enter honey as a contaminant during the beekeeping and honey handling [103]. On the other hand, the detection of taxa pathogenic to humans in a metagenomic study is likely overrepresented, as such microbes are thoroughly sequenced and thus better presented in reference databases in comparison to all other microbes. This may cause a bias in taxonomic assignment [104–106].

While the above findings point to some pathogen spreading by honeybees, it should not be assumed that honeybees would be the only pollinators spreading pathogenic, as well as beneficial, microbes while pollinating and moving around in their surroundings. The strength of functions and the identities of associations will depend on the pollinators’ preferences and behaviours. Nonetheless, honeybees are the most abundant pollinators globally [6, 7], and therefore their actions come with leveraged impacts.

### The honeybee itself serves as a home for a range of bacteria

Honeybees, and their hives, are known to host a set of microbes. Honeybee is a model in development for gut microbe studies, thus these gut microbes are well sequenced [107], enabling their identification based on DNA. The five core bacteria of honeybee’s gut, *Lactobacillus kunkeei*, *L*. Firm-4 and Firm-5, *Gilliamella*, *Snodgrasella* and *Bifidobacterium* [44–47, 107, 108] are rarely detected in any other environment than bees’ guts [109]. Yet, their DNA is relatively abundant in our honey samples. The high consistency of the gut microbiota suggests they are all central to the honeybee’s welfare [110], making hosting them crucial and beneficial for the bees. Apart from the core set, many of the other common bee gut microbes, as *Acinetobacter*, *Bartonella*, *Bacillus*, *Lactococcus*, *Frischella*, *Enterobacter* and *Commensalibacter*, may have both beneficial and detrimental effects on honeybees themselves and on other taxa [44, 45, 55, 64, 65, 111–115]. While honeybees act as hosts for these taxa, this association may not be reflected in any true ecosystem service. Hosting them might not improve the honeybees’ wealth, thus it might not lead to better performance in pollination or in honey production. The same goes for beehive microbes. Some of them are clearly beneficial, like *Parasaccharibacter apium*, which increases a colony’s resistance to infection by *Nosema* species, one of the most common causes of colony losses [116]. By comparison, *Zygosaccharomyces mellis*, *Z*. *rouxii* and *Saccharomyces* are yeasts tolerant of high sugar contents and thus able to grow in moist honey [117, 118], causing the fermentation of honey [118].

## Conclusions

Describing a species’ functional niche requires the measurement of its actions in an ecosystem or in a specific region, which is no trivial task. Yet, using information on what taxa a species associates with, and with what frequency and strength such interactions or contacts occur, can inform us about the species’ role in the community and ecosystem. Using the honeybee as a model species, we have shown how DNA in honey can be used in defining the functional niche of a specific species in a case region (Northern Europe), and pointed to ecosystem services and disservices likely emanating from these functions. The same approaches are likely to prove fruitful in a much wider context. With a set of samples of honey, of the organisms themselves, or of their feeding substrates (like flowers; [119]) from a region, a season, or a habitat type, one may be able to characterize the full span of interactions, the resulting functions, and the ultimate ecosystem services and disservices accrued from these functions. The resulting information can guide us to examine important associations in more detail, in search of improving the ratio of ecosystem services to disservices provided by honeybees or by other taxa in concern. Beyond insights into the general niche of the species, such approaches may also illuminate the spatiotemporal spread of particular associations, such as the regional spread of a pathogen, or a switch in host use under novel biotic conditions [120]. While the causality of the associations reported here remain to be established, our study offers testable, data-driven hypotheses regarding the associations, services and disservices sustained by organisms across the world.

## Supporting information

S1 Text Methods for metagenomics and DNA metabarcoding. (DOCX)

S2 Text Assessment of frequency of occurrence of genera in the honey samples from one beekeeper and from retail market. (DOCX)

S1 Fig Map of the honey sample origins. Map showing the origin of the honey samples from Northern Europe. The small honeycombs show samples that were obtained directly from one beekeeper and the large honeycombs show samples obtained from retail market, likely compassing honey from more than one beekeeper. Note that many of the samples’ origins overlap. The map was created with the program QGIS, version 3.10.4 (https://qgis.org/).
(TIF)

S2 Fig Frequency of occurrence of genera in honey samples obtained from an individual beekeeper vs samples obtained from thebretail market. Honey samples obtained from a single beekeeper and samples obtained from the retail market provided highly similar estimates of the genus-specific occurrence of taxa. Shown is the frequency of occurrence (%FOO) per genus among samples obtained from the retail market (x-axis) vs from a single beekeeper (y-axis). The higher was the estimate from the former, the higher was also the latter (Pearson *r* = 0.87, *n* = 88 taxa, p<0.00001). To avoid spurious correlations, we here include only those bacterial (38 genera; shown in black), fungal (6 genera; shown in blue) and plant (44 genera; shown in green) genera which occurred at a mean relative read abundance (mean RRA) exceeding 0.01% across samples. To show overlapping data points, data points have been jittered in both the vertical and horizontal dimension by up to 4 units of %FOO. For visual comparison, the dotted line shows a hypothetical 1:1 relation obtained if both types of samples yielded exactly the same estimate of %FOO.
(TIF)

S1 Table Primers used for metabarcoding. Primers used for metabarcoding bacteria (16S with two primer pairs, for short called 16Sa [121] and 16Sb [122]), fungi (ITS2 [123]) and plants (ITS2 [124, 125], rbcLa [126, 127] and trnL [128]). The tag part of the primer is shown in small letters and the actual gene region specific primer with capital letters and all primers are given in 5’–3’.
(DOCX)

S2 Table The most abundant bacterial genera from metagenomics and metabarcoding. The hundred most abundant bacterial genera based on their mean RRA from metagenomics, with their %FOO, compared with the mean RRA and %FOO from 16S metabarcoding by the two primer pairs 16Sa and 16Sb.
(DOCX)

S3 Table The most abundant fungal genera from metagenomics and metabarcoding. The hundred most abundant fungal genera based on their mean RRA from metagenomics, with their %FOO, compared with the mean RRA and %FOO from ITS2 metabarcoding.
(DOCX)

S4 Table The most abundant plant genera from metagenomics and metabarcoding. The hundred most abundant plant genera based on their mean RRA from metagenomics, with their %FOO, compared with the mean RRA and %FOO from ITS2, rbcLa and trnL metabarcoding.
(DOCX)

S5 Table Functional classes of the bacterial OTUs from 16S metabarcoding. Functional classes of 16S bacterial OTU with the mean RRA (±SD) and FOO%. The functionality classes are ordered in the table based on the mean RRA from the primers 16Sa, following those from 16Sb. Shown are classes presented by >0.01% of mean RRA.
(DOCX)

S6 Table Functional classes of the fungal genera from ITS2 metabarcoding. Functional classes to which the metabarcoding ITS2 fungal reads were assigned to, with both the mean RRA (±SD) and FOO% of the functionalities. The functionality classes are ordered based on the mean RRA.
(DOCX)
