# Supplementary material for: Reconstructing the ecosystem context of a species: Honey-borne DNA reveals the roles of the honeybee
Source: PLoS One. 2022 Jul 13;17(7):e0268250. doi: 10.1371/journal.pone.0268250 (PMC9278776; doi:10.1371/journal.pone.0268250)
Supplement: S1 Text — (DOCX) [file pone.0268250.s001.docx]

**S1 Text. Assessment of frequency of occurrence of genera in the honey samples from one beekeeper and from retail market**

To assess whether there is a difference in the frequency of occurrence between honey samples obtained directly from beekeepers (15 samples) and the ones from retail market (28 samples), we considered the bacterial, fungal and plant genera with ≥0.01% mean RRA across samples based on metagenomics. Considering honey samples from retail market likely to be honey mixed from more than one beekeeper, we wanted to assess if this would increase the frequency of occurrence of taxa in those samples compared to the samples from an individual beekeeper. We compared the %FOO among these two sample types and found them to be comparable (Fig. S2), with the mean %FOO of honey samples from one beekeeper being slightly higher (91.21%, SD 17.65) than the mean %FOO of honey samples from retail market (88.92%, SD 15.30). This was also the case when considering the bacteria, fungi and plants separately. For bacterial genera the mean %FOO for honey samples from one beekeeper was 88.60 (SD 17.23) while from retail market 87.12% (SD 13.82). For fungal and plant genera the mean %FOO were 82.22 (SD 24.85) and 94.70 (SD 15.93) for samples from one beekeeper, and 79.17 (SD 31.50) and 91.80 (SD 11.98), respectively.

Based on this, the possible mixing of honeys from different beekeepers in the samples from retail market does not cause the frequency of occurrence of taxa to be higher for this set of samples in our study. Yet, unfortunately, for the samples in this study, we do not have data on whether the honey directly from beekeepers originates from one or multiple sites of hives. Likewise, the honey from retail market may originate from one or from multiple beekeepers and sites, but it is more likely to be from more than one beekeeper. Therefore our assessment on the effect of mixing honeys from multiple beekeepers on the frequency of occurrence of taxa is not clear cut, and should be considered in sampling in future studies.
