# Supplementary material for: Reconstructing the ecosystem context of a species: Honey-borne DNA reveals the roles of the honeybee
Source: PLoS One. 2022 Jul 13;17(7):e0268250. doi: 10.1371/journal.pone.0268250 (PMC9278776; doi:10.1371/journal.pone.0268250)
Supplement: S2 Text — (DOCX) [file pone.0268250.s002.docx]

**S2 Text. Methods for metagenomics and DNA metabarcoding**

**DNA extraction, amplification and sequencing**

We use here the data as it was for the previous study [1]. Two 20 g samples from each honey sample were taken to ensure a sufficient amount of DNA for the analyses. One DNA sample was prepared to be used for metagenomics and one for metabarcoding [1]. For each DNA sample, two subsamples, each of 10 g of honey, were diluted to 30 ml of DNA-free water (Lonza, AccuGENE Molecular Biology Water). The subsamples were placed to an oven in +60 °C for 60 min to let the honey to dissolve. The subsamples were centrifuged for 60 min in 8000 g (Centrifuge 5810 R, Eppendorf, Germany) to collect all the tissue material into a pellet. After discarding most of the supernatant, the pellets from the two subsamples were combined into a 2 ml tube, thus generating two samples per each original honey sample. These were further centrifuged for 5 min in 11 000 g (Heraeus Pico 21 centrifuge, Thermo Scientific, USA). Rest of the supernatant was removed.

The pellets were kept in -20 °C until DNA extraction, and the total DNA was extracted with DNeasy Plant Mini Kits (Qiagen, Germany), with the following modifications to the protocol. First, the pellet was resuspended in 400µl of buffer AP1, with 4µl RNase and 4µl proteinase K (20mg/ml, Macherey-Nagel). To break the tissue, e.g. pollen spores, one 3 mm tungsten carbide bead was added to each sample tube and the samples were then disrupted for 2 x 2 min with 30/ rpm (Mixer Mill MM 400, Retsch, Germany). DNA extraction then followed the protocol of the kit, except that we omitted the QIAshredder column step as well as the second washing step, with the buffer AW2, to minimize the loss of DNA.

To identify all the DNA contents in the sample by metagenomics, the DNA was fragmented to 150 bp pieces and prepared into a sequencing library with NexteraXT Kit DNA Library Preparation Kit (Illumina, Inc.), and sequenced in an Illumina NextSeq 500 Sequencer Mid Output (2x150 bp) run. All sequencing was performed by the Functional Genomics Unit [2] at the University of Helsinki, Finland.

To identify bacterial, fungal and plant taxa with DNA from the samples based on metabarcoding, targeted gene regions (Table S1) were amplified as follows. The bacterial and fungal gene regions selected are the same as in worldwide research campaigns [3,4], with the same relatively universal primer pairs [5]; 16S (with two primer set referred to as 16Sa and 16Sb) for bacteria, and ITS2 for fungi. To examine the plant DNA contents in honey samples, the same approach of commonness, universality, and practicality was used to choose the primers targeting three gene regions (ITS2, rbcLa and trnL) [1]. All the gene regions were amplified twice to minimize the bias of initial amplification, in the volume of 15 μl, with 7.5 μl MyTaq Red Mix (Bioline, London, UK), 4.6 μl DNA- and RNA-free water, 0.45 μl of each primer (10 μM) and 2 μl of DNA extract. Amplification was done with an initial denaturation of 5 min at 95 °C, followed by 35 cycles of consisting of denaturation for 40 s in 95 °C, annealing for 60 s in 50 °C for 16Sb, ITS2 for fungi and trnL and in 55 °C for 16Sa, rbcLa and ITS2 for plants and extension for 30 s 72 °C, and after the cycles ending with final extension for 5 min at 72 °C. All the amplicons were checked on a 1% agarose gel. If a reaction had not produced a clear band, the PCR was repeated. The successful PCR replicates were combined before the library-PCR. Illumina‐specific adapters and unique dual‐index combinations for each sample were used [6]. The library PCR was done in a total volume of 10 μl with 5 μl MyTaq Red Mix (Bioline, London, UK), 1.2 μl of each primer (2.5 μM) and 2.6 μl of the locus-specific 1^st^ PCR product. PCR program was the same for all gene regions for the library PCR with an initial denaturation of 4 min in 95 °C, followed by 15 cycles of denaturation for 20 s in 98 °C, annealing for 15 s in 60 °C and extension for 30 s in 72 °C, and ending with a final extension for 3 min in 72 °C. DNA libraries were pooled per gene region and purified using a SPRI bead protocol [7]. The DNA concentration of the cleaned pools were measured with Qubit 2.0 (dsHS DNA Kit, ThermoFisher Scientific). The libraries were sequenced in three MiSeq runs as follows. The two 16S rRNA pools (16Sa and 16Sb) and the fungal ITS pool were combined in equimolar ratios into one run and the *rbc*L*a* and plant ITS2 pools into another, and these were run with v3 chemistry with 300 cycles and 2 x 300 bp paired-end read length. The gene region *trnL* for plants was sequenced on Illumina MiSeq Nano run with v2 chemistry and 2x250 cycles.

In the laboratory all the steps before the amplification of DNA were done in a laminar hood wiped with ethanol and cleaned of DNA with 1 hour UV light every night. Also, we only used DNA-free tubes, pipet tips and PCR plates as well as DNA-free water. To detect possible contamination, we added blank controls to all the DNA extraction and PCR batches, and sequenced these along with the other samples as well as a blank DNA extraction control also for the metagenomics sequencing.

**Bioinformatic processing of sequences**

In regard to the bioinformatics processing of sequence reads, we use here the data as it was processed for the previous study [1]. For the metagenomics sequencing, the bioinformatic processing of reads was done at University of Helsinki’s Biomedicum Functional Genomics Unit [2]. Overall quality of the sequencing was checked with FASTQC and light quality trimming was performed with trimmomatic [8]. After the reads had passed quality control, taxonomic labels were assigned to sequencing reads using Kraken2 [9]. Kraken2 was run against custom-built National Center for Biotechnology Information (NCBI) [10] non-redundant nucleotide database (NT) in September 2019. The database was built limiting Kraken2 hash table size to 100GB. To obtain abundance estimates for different species, Kraken2 report files were used as an input for Bracken [11]. Kraken2 results were examined and combined in Pavian [12]. Further filtering of the reads assigned to families, genera and species was done as follows: the number of reads found in the control sample was subtracted from all the samples for each taxon and all taxa from a sample with less than 20 reads for that taxon were removed.

For the metabarcoding sequences of bacteria, fungi and plants, the bioinformatic processing was done as follows. First, paired ends for each gene region were merged using PEAR [13] with a minimum overlap of 10 base pairs (bp) and a minimum assembly length of 50 bp. The merged reads were only retained if they contained the expected primers at each end. Primers were then removed using ‘Split_on_Primer.py’ (github:Y-Lammers/Split_on_Primer) before cleaning and filtering using PRINSEQ with a minimum mean quality score of 26 and a minimum length of 50bp [14]. Dereplication was done using VSEARCH [15], and the removal of singletons, clustering to OTUs at 97% and mapping of reads against OTUs using UPARSE [16]. Taxonomic assignations were made using RDP [17] for all other gene regions but *trn*L, by comparison against a specific reference databases for each gene region. Reference databases were accessed in April 2019. Specifically, ITS2 and *rbc*L*a* for plants were compared against an ITS2 and a *rbc*L*a* reference databases, respectively [18,19], ITS for fungi against the UNITE fungal ITS reference database, version 8 [20] and 16Sa and 16Sb for bacteria against the 16S rRNA reference database, release 11 [21]. For *trn*L, the taxonomic assignations were made using blastn [22] against the NCBI reference database, release 230 [23] followed by the lowest common ancestor (LCA) analysis, assigning each read to the lowest common ancestor of the set of taxa that it hit in the NCBI database, in MEGAN [24]. To remove possible misassigned reads and false positives, due to tag jumping or contamination, we followed a conservative approach with three steps to further filter all reads and OTUs (following e.g. [25,26]). As small numbers of reads were found in all controls, we subtracted the maximum number of reads for a negative sample from all the samples for each OTU. All samples with fewer than 50 reads in total were removed. Further, all OTUs from a sample with less than 20 reads for the out, or with less than 0.05% of the total read number (all reads assigned to OTUs) of the sample were removed.

**References**

1. Wirta H, Abrego N, Miller K, Roslin T, Vesterinen E. DNA traces the origin of honey by identifying plants, bacteria and fungi. Sci Rep. 2021;11: 4798. doi:10.1038/s41598-021-84174-0

2. Functional Genomics Unit, University of Helsinki, Finland. Available: www.helsinki.fi/en/infrastructures/genome-analysis/biomedicum-functional-genomics-unit

3. Caporaso JG, Lauber CL, Walters WA, Berg-Lyons D, Lozupone CA, Turnbaugh PJ, et al. Global patterns of 16S rRNA diversity at a depth of millions of sequences per sample. Proc Natl Acad Sci U S A. 2011;108: 4516–4522. doi:10.1073/pnas.1000080107

4. Schoch CL, Seifert KA, Huhndorf S, Robert V, Spouge JL, Levesque CA, et al. Nuclear ribosomal internal transcribed spacer (ITS) region as a universal DNA barcode marker for Fungi. Proc Natl Acad Sci U S A. 2012;109: 6241–6246. doi:10.1073/pnas.1117018109

5. Põlme S, Abarenkov K, Henrik Nilsson R, Lindahl BD, Clemmensen KE, Kauserud H, et al. FungalTraits: a user-friendly traits database of fungi and fungus-like stramenopiles. Fungal Divers. 2020;105. doi:10.1007/s13225-020-00466-2

6. Vesterinen EJ, Puisto AIE, Blomberg AS, Lilley TM. Table for five, please: Dietary partitioning in boreal bats. Ecol Evol. 2018;8: 10914–10937. doi:10.1002/ece3.4559

7. Vesterinen EJ, Ruokolainen L, Wahlberg N, Peña C, Roslin T, Laine VN, et al. What you need is what you eat? Prey selection by the bat *Myotis daubentonii*. Mol Ecol. 2016;25: 1581–1594. doi:10.1111/mec.13564

8. Bolger AM, Lohse M, Usadel B. Genome analysis Trimmomatic: a flexible trimmer for Illumina sequence data. 2014;30: 2114–2120. doi:10.1093/bioinformatics/btu170

9. Wood DE, Lu J, Langmead B. Improved metagenomic analysis with Kraken 2. Genome Biol. 2019;20. doi:10.1186/s13059-019-1891-0

10. National Center for Biotechnology Information (NCBI); Bethesda (MD): National Library of Medicine (US). 1988. Available: https://www.ncbi.nlm.nih.gov/

11. Lu J, Breitwieser FP, Thielen P, Salzberg SL. Bracken: Estimating species abundance in metagenomics data. PeerJ Comput Sci. 2017;2017: e104. doi:10.7717/peerj-cs.104

12. Breitwieser FP, Salzberg SL. Pavian: Interactive analysis of metagenomics data for microbiome studies and pathogen identification. Bioinformatics. 2020;36: 1303–1304. doi:10.1093/bioinformatics/btz715

13. Zhang J, Kobert K, Flouri T, Stamatakis A. PEAR: A fast and accurate Illumina Paired-End reAd mergeR. Bioinformatics. 2014;30: 614–620. doi:10.1093/bioinformatics/btt593

14. Schmieder R, Edwards R. Quality control and preprocessing of metagenomic datasets. Bioinformatics. 2011;27: 863–864.

15. Rognes T, Flouri T, Nichols B, Quince C, Mahé F. VSEARCH: a versatile open source tool for metagenomics. PeerJ. 2016. doi:10.7717/peerj.2584

16. Edgar RC. UPARSE: Highly accurate OTU sequences from microbial amplicon reads. Nat Methods. 2013;10: 996–998. doi:10.1038/nmeth.2604

17. Wang Q, Garrity GM, Tiedje JM, Cole JR. Naïve Bayesian classifier for rapid assignment of rRNA sequences into the new bacterial taxonomy. Appl Environ Microbiol. 2007;73: 5261–5267. doi:10.1128/AEM.00062-07

18. Sickel W, Ankenbrand MJ, Grimmer G, Holzschuh A, Härtel S, Lanzen J, et al. Increased efficiency in identifying mixed pollen samples by meta-barcoding with a dual-indexing approach. BMC Ecol. 2015;15: 1–9. doi:10.1186/s12898-015-0051-y

19. Bell KL, Loeffler VM, Brosi BJ. An rbcL Reference Library to Aid in the Identification of Plant Species Mixtures by DNA Metabarcoding . Appl Plant Sci. 2017;5: 1600110. doi:10.3732/apps.1600110

20. Nilsson RH, Larsson K-H, Taylor AFS, Bengtsson-Palme J, Jeppesen TS, Schigel D, et al. The UNITE database for molecular identification of fungi: handling dark taxa and parallel taxonomic classifications. Nucleic Acids Res. 2018;47: 259–264. doi:10.1093/nar/gky1022

21. Cole JR, Wang Q, Cardenas E, Fish J, Chai B, Farris RJ, et al. The Ribosomal Database Project: improved alignments and new tools for rRNA analysis. Nucleic Acids Res. 2008;37: 141–145. doi:10.1093/nar/gkn879

22. Altschul SF, Gish W, Miller W, Myers EW, Lipman DJ. Basic local alignment search tool. J Mol Biol. 1990;215: 403–410. doi:10.1016/S0022-2836(05)80360-2

23. Benson DA, Karsch-Mizrachi I, Lipman DJ, Ostell J, Sayers EW. GenBank. Nucleic Acids Res. 2011;39. doi:10.1093/nar/gkq1079

24. Huson DH, Auch AF, Qi J, Schuster SC. MEGAN analysis of metagenomic data. Genome Res. 2007;17: 377–386. doi:10.1101/gr.5969107

25. Lee T, Alemseged Y, Mitchell A. Dropping Hints: Estimating the diets of livestock in rangelands using DNA metabarcoding of faeces. Metabarcoding and Metagenomics. 2018;2: e22467. doi:10.3897/mbmg.2.22467

26. Alberdi A, Garin I, Aizpurua O, Aihartza J. The foraging ecology of the Mountain Long-eared bat Plecotus macrobullaris revealed with DNA mini-barcodes. PLoS One. 2012;7. doi:10.1371/journal.pone.0035692
