# Supplementary figures and images for: Reconstructing the ecosystem context of a species: Honey-borne DNA reveals the roles of the honeybee
Source: PLoS One. 2022 Jul 13;17(7):e0268250. doi: 10.1371/journal.pone.0268250 (PMC9278776; doi:10.1371/journal.pone.0268250)

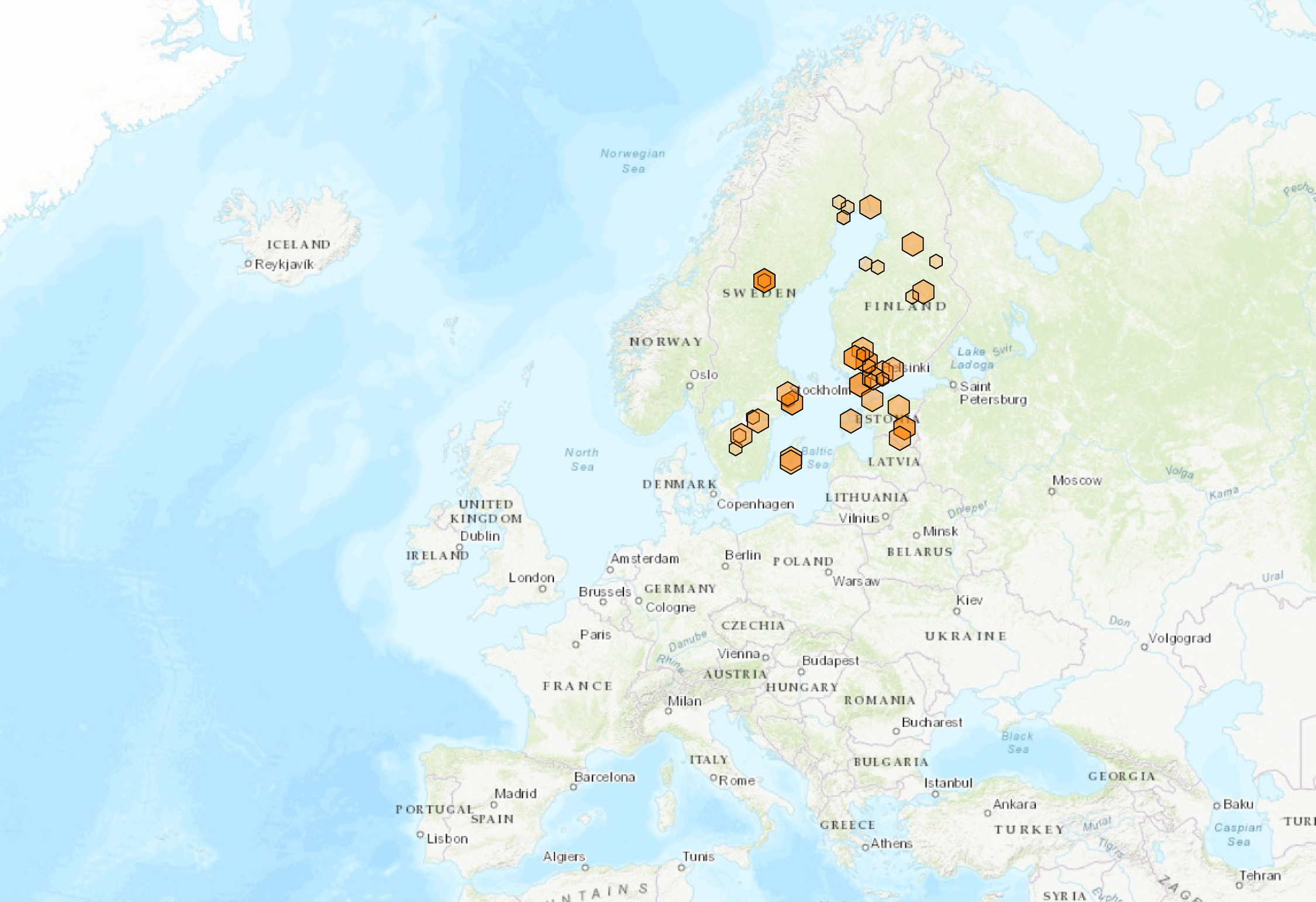

Supplement: S1 Fig — Map showing the origin of the honey samples from Northern Europe. The small honeycombs show samples that were obtained directly from one beekeeper and the large honeycombs show samples obtained from retail market, likely compassing honey from more than one beekeeper. Note that many of the samples’ origins overlap. The map was created with the program QGIS, version 3.10.4 (https://qgis.org/). (TIF) [file pone.0268250.s003.tif]

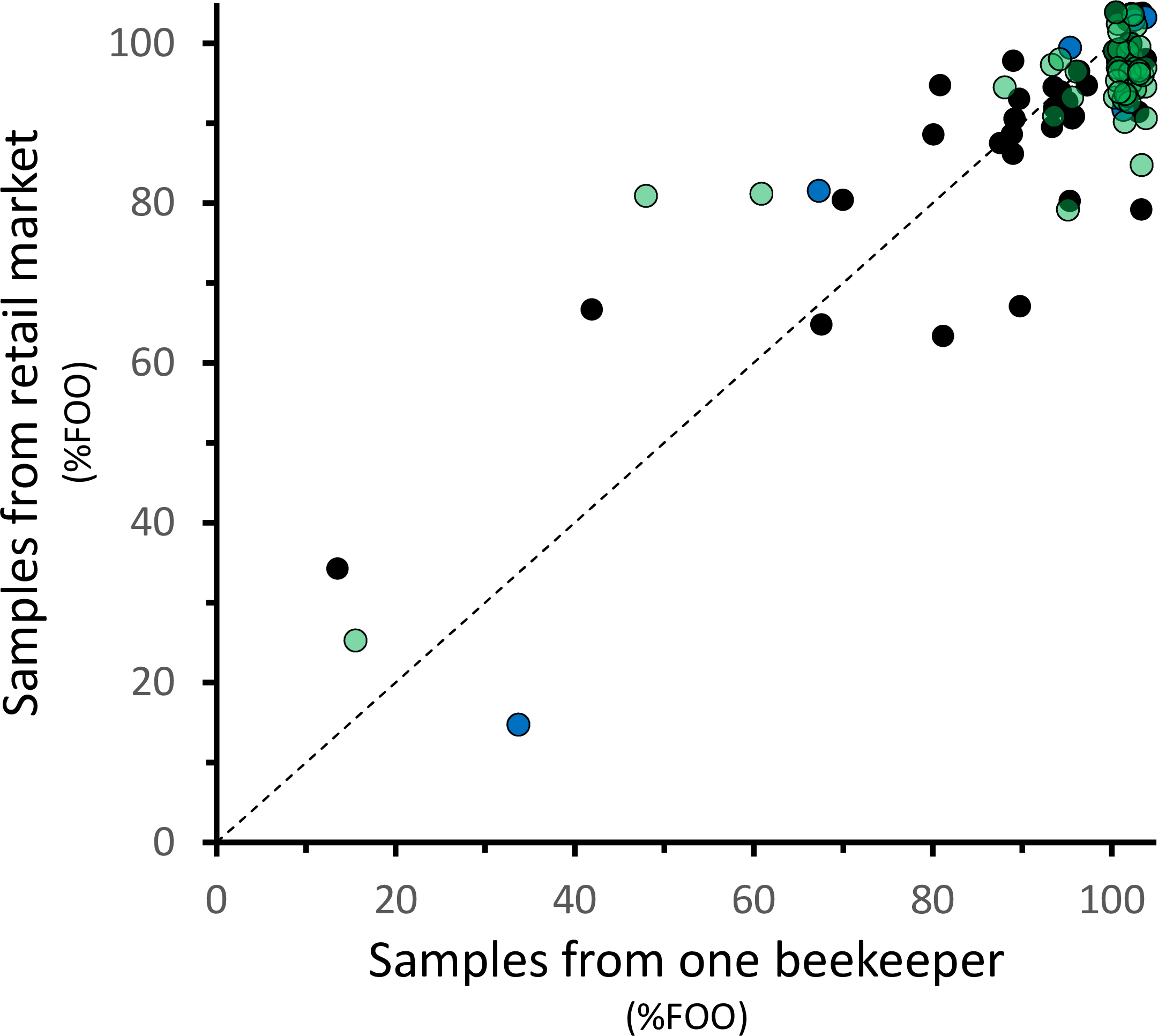

Supplement: S2 Fig — Honey samples obtained from a single beekeeper and samples obtained from the retail market provided highly similar estimates of the genus-specific occurrence of taxa. Shown is the frequency of occurrence (%FOO) per genus among samples obtained from the retail market (x-axis) vs from a single beekeeper (y-axis). The higher was the estimate from the former, the higher was also the latter (Pearson r = 0.87, n = 88 taxa, p<0.00001). To avoid spurious correlations, we here include only those bacterial (38 genera; shown in black), fungal (6 genera; shown in blue) and plant (44 genera; shown in green) genera which occurred at a mean relative read abundance (mean RRA) exceeding 0.01% across samples. To show overlapping data points, data points have been jittered in both the vertical and horizontal dimension by up to 4 units of %FOO. For visual comparison, the dotted line shows a hypothetical 1:1 relation obtained if both types of samples yielded exactly the same estimate of %FOO. (TIF) [file pone.0268250.s004.tif]
