# Supplementary material for: Reconstructing the ecosystem context of a species: Honey-borne DNA reveals the roles of the honeybee
Source: PLoS One. 2022 Jul 13;17(7):e0268250. doi: 10.1371/journal.pone.0268250 (PMC9278776; doi:10.1371/journal.pone.0268250)
Supplement: S1 Table — Primers used for metabarcoding bacteria (16S with two primer pairs, for short called 16Sa [121] and 16Sb [122]), fungi (ITS2 [123]) and plants (ITS2 [124, 125], rbcLa [126, 127] and trnL [128]). The tag part of the primer is shown in small letters and the actual gene region specific primer with capital letters and all primers are given in 5’–3’. (DOCX) [file pone.0268250.s005.docx]

**S1 Table. Primers used for metabarcoding**

Primers used for metabarcoding bacteria (16S with two primer pairs, for short called 16Sa [27] and 16Sb [28]), fungi (ITS2 [29]) and plants (ITS2 [30,31], rbcLa [32,33] and trnL [34]). The tag part of the primer is shown in small letters and the actual gene region specific primer with capital letters. All primers are given in the 5' - 3' direction.

| Gene region | Primer | Primer sequence with tags |
| --- | --- | --- |
| 16Sa | tagF_16S_515FB | tcgtcggcagcgtcagatgtgtataagagacagGTGYCAGCMGCCGCGGTAA |
|  | tagR_16S_806RB | gtctcgtgggctcggagatgtgtataagagacagGGACTACNVGGGTWTCTAAT |
| 16Sb | tagF_16S_F-341F | tcgtcggcagcgtcagatgtgtataagagacagCCTACGGGNGGCWGCAG |
|  | tagR_16S_R-805R | gtctcgtgggctcggagatgtgtataagagacagGACTACHVGGGTATCTAATCC |
| ITS2 | tagF_ITS3_KYO2 | tcgtcggcagcgtcagatgtgtataagagacagAHCGATGAAGAACRYAG |
| (fungi) | tagR_ITS4_KYO3 | gtctcgtgggctcggagatgtgtataagagacagCTBTTVCCKCTTCACTCG |
| ITS2 | tagF_ITS2-F | tcgtcggcagcgtcagatgtgtataagagacagATGCGATACTTGGTGTGAAT |
| (plants) | tagR_ITS2-R | gtctcgtgggctcggagatgtgtataagagacagTCCTCCGCTTATTGATATGC |
| *rbc*L*a* | tagF_rbcLa-F | tcgtcggcagcgtcagatgtgtataagagacagATGTCACCACAAACAGAGACTAAAGC |
|  | tagR_rbcLa-R | gtctcgtgggctcggagatgtgtataagagacagCGGTCCAYACAGYBGTCCAKGTACC |
| *trn*L | tagF_trnL-c | tcgtcggcagcgtcagatgtgtataagagacagCGAAATCGGTAGACGCTACG |
|  | tagR_trnL-h | gtctcgtgggctcggagatgtgtataagagacagCCATTGAGTCTCTGCACCTATC |
