# Supplementary material for: Reconstructing the ecosystem context of a species: Honey-borne DNA reveals the roles of the honeybee
Source: PLoS One. 2022 Jul 13;17(7):e0268250. doi: 10.1371/journal.pone.0268250 (PMC9278776; doi:10.1371/journal.pone.0268250)
Supplement: S2 Table — The hundred most abundant bacterial genera based on their mean RRA from metagenomics, with their %FOO, compared with the mean RRA and %FOO from 16S metabarcoding by the two primer pairs 16Sa and 16Sb. (DOCX) [file pone.0268250.s006.docx]

**S2 Table. The most abundant bacterial genera from metagenomics and metabarcoding**

The hundred most abundant bacterial genera based on their mean RRA from metagenomics, with their %FOO, compared with the mean RRA and %FOO from 16S metabarcoding by the two primer pairs 16Sa and 16Sb.

|  | metagenomics | |  |  | 16sa |  |  |  | 16Sb |  |  |
| --- | --- | --- | --- | --- | --- | --- | --- | --- | --- | --- | --- |
|  | RRA |  | %FOO |  | RRA |  | %FOO |  | RRA |  | %FOO |
| genus | mean | SD |  |  | mean | SD |  |  | mean | SD |  |
| *Lactobacillus* | 13.714 | 17.52 | 97.67 |  | 44.436 | 25.57 | 91.30 |  | 20.511 | 24.46 | 82.61 |
| *Pseudomonas* | 0.318 | 0.48 | 93.02 |  | 1.646 | 1.68 | 80.43 |  | 0.486 | 0.63 | 60.87 |
| *Parasaccharibacter* | 0.248 | 0.32 | 97.67 |  | na | na | na |  | na | na | na |
| *Pantoea* | 0.189 | 0.32 | 97.67 |  | na | na | na |  | 0.346 | 1.35 | 30.43 |
| *Rickettsia* | 0.170 | 0.70 | 93.02 |  | 0.131 | 0.39 | 26.09 |  | 0.034 | 0.12 | 8.70 |
| *Erwinia* | 0.118 | 0.14 | 97.67 |  | 5.111 | 10.12 | 76.09 |  | na | na | na |
| *Acinetobacter* | 0.105 | 0.24 | 86.05 |  | 4.851 | 8.20 | 84.78 |  | 1.511 | 4.35 | 47.83 |
| *Mesoplasma* | 0.090 | 0.30 | 74.42 |  | 1.972 | 6.14 | 50.00 |  | 1.950 | 7.04 | 17.39 |
| *Bartonella* | 0.074 | 0.29 | 90.70 |  | na | na | na |  | na | na | na |
| *Serratia* | 0.068 | 0.15 | 95.35 |  | 0.099 | 0.26 | 23.91 |  | 0.099 | 0.31 | 19.57 |
| *Entomoplasma* | 0.058 | 0.19 | 65.12 |  | na | na | na |  | na | na | na |
| *Arsenophonus* | 0.056 | 0.11 | 88.37 |  | 1.184 | 3.11 | 63.04 |  | 0.789 | 3.49 | 21.74 |
| *Spiroplasma* | 0.056 | 0.08 | 95.35 |  | 0.050 | 0.10 | 28.26 |  | 0.009 | 0.06 | 2.17 |
| *Streptomyces* | 0.052 | 0.03 | 100.00 |  | na | na | na |  | na | na | na |
| *Gilliamella* | 0.049 | 0.17 | 83.72 |  | na | na | na |  | na | na | na |
| *Rahnella* | 0.048 | 0.14 | 97.67 |  | na | na | na |  | na | na | na |
| *Bacillus* | 0.039 | 0.03 | 100.00 |  | 0.495 | 1.63 | 36.96 |  | 2.164 | 4.00 | 50.00 |
| *Lactococcus* | 0.030 | 0.06 | 93.02 |  | 0.195 | 0.43 | 45.65 |  | 0.021 | 0.10 | 6.52 |
| *Snodgrassella* | 0.027 | 0.06 | 88.37 |  | 0.064 | 0.25 | 6.52 |  | 0.011 | 0.05 | 4.35 |
| *Melissococcus* | 0.025 | 0.07 | 55.81 |  | na | na | na |  | 14.077 | 25.07 | 32.61 |
| *Frischella* | 0.022 | 0.06 | 83.72 |  | 0.063 | 0.10 | 34.78 |  | 0.004 | 0.03 | 2.17 |
| *Streptococcus* | 0.021 | 0.02 | 90.70 |  | 0.019 | 0.09 | 6.52 |  | 0.008 | 0.05 | 2.17 |
| *Enterobacter* | 0.020 | 0.03 | 100.00 |  | 0.477 | 0.73 | 39.13 |  | na | na | na |
| *Commensalibacter* | 0.019 | 0.02 | 97.67 |  | na | na | na |  | na | na | na |
| *Clostridium* | 0.019 | 0.02 | 93.02 |  | 0.742 | 1.59 | 41.30 |  | 2.970 | 8.25 | 58.70 |
| *Lonsdalea* | 0.016 | 0.07 | 25.58 |  | 0.417 | 1.08 | 17.39 |  | 0.058 | 0.35 | 4.35 |
| *Staphylococcus* | 0.015 | 0.02 | 72.09 |  | na | na | na |  | na | na | na |
| *Paenibacillus* | 0.015 | 0.01 | 100.00 |  | 1.243 | 3.24 | 41.30 |  | 3.543 | 9.93 | 47.83 |
| *Yersinia* | 0.015 | 0.03 | 86.05 |  | na | na | na |  | 0.018 | 0.09 | 4.35 |
| *Enterococcus* | 0.014 | 0.02 | 88.37 |  | 4.391 | 13.87 | 10.87 |  | na | na | na |
| *Vibrio* | 0.014 | 0.02 | 90.70 |  | na | na | na |  | na | na | na |
| *Klebsiella* | 0.014 | 0.02 | 90.70 |  | na | na | na |  | na | na | na |
| *Leuconostoc* | 0.013 | 0.02 | 90.70 |  | 0.012 | 0.03 | 10.87 |  | na | na | na |
| *Burkholderia* | 0.012 | 0.01 | 100.00 |  | 0.036 | 0.09 | 17.39 |  | 0.003 | 0.02 | 2.17 |
| *Mycoplasma* | 0.012 | 0.02 | 93.02 |  | 0.002 | 0.01 | 2.17 |  | na | na | na |
| *Bifidobacterium* | 0.010 | 0.02 | 86.05 |  | 0.099 | 0.20 | 26.09 |  | 0.058 | 0.19 | 13.04 |
| *Proteus* | 0.010 | 0.03 | 67.44 |  | 0.013 | 0.05 | 6.52 |  | na | na | na |
| *Escherichia* | 0.010 | 0.01 | 83.72 |  | na | na | na |  | na | na | na |
| *Weissella* | 0.009 | 0.01 | 86.05 |  | 0.007 | 0.04 | 4.35 |  | na | na | na |
| *Pediococcus* | 0.009 | 0.01 | 86.05 |  | na | na | na |  | na | na | na |
| *Citrobacter* | 0.009 | 0.02 | 86.05 |  | na | na | na |  | na | na | na |
| *Paraburkholderia* | 0.009 | 0.01 | 95.35 |  | na | na | na |  | na | na | na |
| *Salmonella* | 0.009 | 0.01 | 90.70 |  | na | na | na |  | na | na | na |
| *Shewanella* | 0.009 | 0.01 | 81.40 |  | na | na | na |  | 0.006 | 0.04 | 2.17 |
| *Acetobacter* | 0.008 | 0.02 | 81.40 |  | 0.003 | 0.02 | 2.17 |  | na | na | na |
| *Providencia* | 0.007 | 0.01 | 79.07 |  | na | na | na |  | na | na | na |
| *Tatumella* | 0.007 | 0.02 | 58.14 |  | na | na | na |  | na | na | na |
| *Pseudoalteromonas* | 0.007 | 0.01 | 81.40 |  | na | na | na |  | na | na | na |
| *Chryseobacterium* | 0.007 | 0.01 | 81.40 |  | 0.009 | 0.04 | 4.35 |  | 0.010 | 0.05 | 4.35 |
| *Carnobacterium* | 0.007 | 0.01 | 86.05 |  | 0.013 | 0.06 | 6.52 |  | 0.039 | 0.20 | 4.35 |
| *Frankia* | 0.006 | 0.01 | 83.72 |  | na | na | na |  | na | na | na |
| *Herbaspirillum* | 0.006 | 0.01 | 83.72 |  | na | na | na |  | na | na | na |
| *Legionella* | 0.006 | 0.01 | 81.40 |  | 0.031 | 0.16 | 8.70 |  | 0.010 | 0.05 | 4.35 |
| *Megasphaera* | 0.006 | 0.01 | 72.09 |  | na | na | na |  | na | na | na |
| *Pectobacterium* | 0.006 | 0.01 | 72.09 |  | na | na | na |  | na | na | na |
| *Fusobacterium* | 0.005 | 0.01 | 86.05 |  | na | na | na |  | 0.004 | 0.03 | 2.17 |
| *Bradyrhizobium* | 0.005 | 0.01 | 60.47 |  | 0.008 | 0.04 | 4.35 |  | 0.011 | 0.04 | 6.52 |
| *Gluconobacter* | 0.005 | 0.01 | 69.77 |  | 0.168 | 0.50 | 15.22 |  | 0.369 | 1.44 | 17.39 |
| *Dickeya* | 0.005 | 0.01 | 76.74 |  | na | na | na |  | na | na | na |
| *Hafnia* | 0.005 | 0.01 | 67.44 |  | 0.009 | 0.04 | 6.52 |  | na | na | na |
| *Cedecea* | 0.005 | 0.01 | 79.07 |  | 0.034 | 0.07 | 26.09 |  | na | na | na |
| *Komagataeibacter* | 0.005 | 0.01 | 79.07 |  | 0.005 | 0.03 | 4.35 |  | 0.026 | 0.18 | 2.17 |
| *Campylobacter* | 0.005 | 0.00 | 88.37 |  | na | na | na |  | na | na | na |
| *Rhizobium* | 0.005 | 0.00 | 93.02 |  | 0.264 | 1.05 | 15.22 |  | na | na | na |
| *Haemophilus* | 0.005 | 0.01 | 72.09 |  | 0.019 | 0.08 | 8.70 |  | na | na | na |
| *Lysinibacillus* | 0.004 | 0.00 | 88.37 |  | 0.418 | 2.27 | 4.35 |  | 1.233 | 3.12 | 45.65 |
| *Buchnera* | 0.004 | 0.01 | 76.74 |  | na | na | na |  | na | na | na |
| *Arcobacter* | 0.004 | 0.01 | 72.09 |  | 0.009 | 0.04 | 4.35 |  | na | na | na |
| *Cronobacter* | 0.004 | 0.01 | 67.44 |  | na | na | na |  | na | na | na |
| *Gynuella* | 0.004 | 0.00 | 76.74 |  | na | na | na |  | na | na | na |
| *Xenorhabdus* | 0.004 | 0.01 | 69.77 |  | na | na | na |  | na | na | na |
| *Nostoc* | 0.004 | 0.00 | 79.07 |  | na | na | na |  | na | na | na |
| *Calothrix* | 0.004 | 0.00 | 86.05 |  | na | na | na |  | na | na | na |
| *Sphingomonas* | 0.004 | 0.01 | 58.14 |  | 0.122 | 0.22 | 36.96 |  | 0.107 | 0.33 | 17.39 |
| *Hydrogenophaga* | 0.004 | 0.00 | 74.42 |  | na | na | na |  | na | na | na |
| *Corynebacterium* | 0.004 | 0.00 | 65.12 |  | 0.051 | 0.23 | 10.87 |  | 0.038 | 0.17 | 8.70 |
| *Xanthomonas* | 0.003 | 0.00 | 69.77 |  | 0.007 | 0.04 | 4.35 |  | na | na | na |
| *Listeria* | 0.003 | 0.00 | 69.77 |  | na | na | na |  | na | na | na |
| *Neisseria* | 0.003 | 0.01 | 55.81 |  | 0.007 | 0.03 | 4.35 |  | 0.004 | 0.02 | 2.17 |
| *Mycobacterium* | 0.003 | 0.00 | 72.09 |  | na | na | na |  | na | na | na |
| *Micropruina* | 0.003 | 0.00 | 72.09 |  | na | na | na |  | na | na | na |
| *Brenneria* | 0.003 | 0.01 | 58.14 |  | na | na | na |  | na | na | na |
| *Helicobacter* | 0.003 | 0.00 | 81.40 |  | na | na | na |  | na | na | na |
| *Candidatus Hamiltonella* | 0.003 | 0.01 | 25.58 |  | na | na | na |  | na | na | na |
| *Kosakonia* | 0.003 | 0.01 | 55.81 |  | na | na | na |  | na | na | na |
| *Arthrobacter* | 0.003 | 0.00 | 69.77 |  | 0.019 | 0.11 | 4.35 |  | na | na | na |
| *Mesorhizobium* | 0.003 | 0.00 | 58.14 |  | na | na | na |  | na | na | na |
| *Neokomagataea* | 0.003 | 0.01 | 58.14 |  | na | na | na |  | na | na | na |
| *Actinomadura* | 0.003 | 0.00 | 81.40 |  | na | na | na |  | na | na | na |
| *Ralstonia* | 0.003 | 0.00 | 65.12 |  | na | na | na |  | na | na | na |
| *Aeromonas* | 0.003 | 0.00 | 62.79 |  | na | na | na |  | na | na | na |
| *Vagococcus* | 0.003 | 0.00 | 62.79 |  | na | na | na |  | na | na | na |
| *Synechococcus* | 0.003 | 0.00 | 93.02 |  | na | na | na |  | na | na | na |
| *Actinomyces* | 0.003 | 0.00 | 88.37 |  | 0.004 | 0.02 | 4.35 |  | 0.005 | 0.02 | 4.35 |
| *Lelliottia* | 0.003 | 0.00 | 62.79 |  | na | na | na |  | na | na | na |
| *Morganella* | 0.002 | 0.01 | 30.23 |  | 0.007 | 0.03 | 4.35 |  | na | na | na |
| *Stenotrophomonas* | 0.002 | 0.00 | 51.16 |  | 0.015 | 0.05 | 8.70 |  | 0.013 | 0.09 | 2.17 |
| *Nitrospirillum* | 0.002 | 0.00 | 67.44 |  | 0.002 | 0.01 | 2.17 |  | na | na | na |
| *Alteromonas* | 0.002 | 0.00 | 55.81 |  | na | na | na |  | na | na | na |
| *Bacteroides* | 0.002 | 0.00 | 65.12 |  | na | na | na |  | na | na | na |
