# Supplementary material for: Reconstructing the ecosystem context of a species: Honey-borne DNA reveals the roles of the honeybee
Source: PLoS One. 2022 Jul 13;17(7):e0268250. doi: 10.1371/journal.pone.0268250 (PMC9278776; doi:10.1371/journal.pone.0268250)
Supplement: S3 Table — The hundred most abundant fungal genera based on their mean RRA from metagenomics, with their %FOO, compared with the mean RRA and %FOO from ITS2 metabarcoding. (DOCX) [file pone.0268250.s007.docx]

**S3 Table. The most abundant fungal genera from metagenomics and metabarcoding**

The hundred most abundant fungal genera based on their mean RRA from metagenomics, with their %FOO, compared with the mean RRA and %FOO from ITS2 metabarcoding.

|  | metagenomics | |  |  | ITS2 |  |  |
| --- | --- | --- | --- | --- | --- | --- | --- |
|  | RRA |  | %FOO | | RRA |  | %FOO |
| genus | mean | SD |  |  | mean | SD |  |
| *Zygosaccharomyces* | 0.653 | 2.541 | 95.3 |  | 18.125 | 27.725 | 76.1 |
| *Claviceps* | 0.051 | 0.324 | 18.6 |  | 1.814 | 7.550 | 13.0 |
| *Saccharomyces* | 0.022 | 0.073 | 100.0 |  | 0.008 | 0.052 | 2.2 |
| *Aspergillus* | 0.017 | 0.015 | 93.0 |  | 0.342 | 1.171 | 34.8 |
| *Naumovozyma* | 0.010 | 0.037 | 74.4 |  | na | na | na |
| *Cryptococcus* | 0.010 | 0.006 | 100.0 |  | 0.019 | 0.054 | 13.0 |
| *Fusarium* | 0.009 | 0.007 | 95.3 |  | 0.006 | 0.041 | 2.2 |
| *Tetrapisispora* | 0.009 | 0.030 | 79.1 |  | na | na | na |
| *Kluyveromyces* | 0.008 | 0.024 | 76.7 |  | na | na | na |
| *Candida* | 0.007 | 0.016 | 79.1 |  | 6.450 | 11.640 | 71.7 |
| *Torulaspora* | 0.007 | 0.026 | 51.2 |  | na | na | na |
| *Aureobasidium* | 0.007 | 0.013 | 100.0 |  | 1.837 | 5.296 | 41.3 |
| *Kazachstania* | 0.006 | 0.022 | 74.4 |  | na | na | na |
| *Vanderwaltozyma* | 0.006 | 0.020 | 62.8 |  | na | na | na |
| *Saccharomycopsis* | 0.006 | 0.009 | 95.3 |  | na | na | na |
| *Metschnikowia* | 0.006 | 0.012 | 83.7 |  | 6.032 | 8.283 | 89.1 |
| *Leptosphaeria* | 0.005 | 0.002 | 100.0 |  | na | na | na |
| *Talaromyces* | 0.005 | 0.004 | 93.0 |  | na | na | na |
| *Rhizophagus* | 0.004 | 0.003 | 95.3 |  | na | na | na |
| *Clavispora* | 0.004 | 0.008 | 86.0 |  | 0.002 | 0.013 | 2.2 |
| *Alternaria* | 0.004 | 0.004 | 95.3 |  | 0.015 | 0.071 | 6.5 |
| *Exophiala* | 0.004 | 0.003 | 90.7 |  | 0.006 | 0.028 | 4.3 |
| *Eremothecium* | 0.004 | 0.014 | 65.1 |  | na | na | na |
| *Wickerhamomyces* | 0.004 | 0.013 | 58.1 |  | na | na | na |
| *Lachancea* | 0.004 | 0.014 | 44.2 |  | na | na | na |
| *Penicillium* | 0.004 | 0.003 | 95.3 |  | 0.833 | 3.008 | 56.5 |
| *Nakaseomyces* | 0.003 | 0.012 | 39.5 |  | na | na | na |
| *Sporisorium* | 0.003 | 0.002 | 93.0 |  | na | na | na |
| *Trichoderma* | 0.003 | 0.003 | 81.4 |  | na | na | na |
| *Hyphopichia* | 0.003 | 0.007 | 81.4 |  | na | na | na |
| *Debaryomyces* | 0.003 | 0.006 | 65.1 |  | 0.034 | 0.196 | 4.3 |
| *Colletotrichum* | 0.003 | 0.004 | 76.7 |  | na | na | na |
| *Sclerotinia* | 0.003 | 0.001 | 100.0 |  | 0.010 | 0.069 | 2.2 |
| *Exserohilum* | 0.003 | 0.003 | 74.4 |  | na | na | na |
| *Blumeria* | 0.003 | 0.002 | 83.7 |  | 0.016 | 0.054 | 10.9 |
| *Botrytis* | 0.002 | 0.002 | 69.8 |  | 0.228 | 0.841 | 19.6 |
| *Pseudogymnoascus* | 0.002 | 0.002 | 74.4 |  | 0.002 | 0.012 | 2.2 |
| *Chaetomium* | 0.002 | 0.003 | 72.1 |  | 0.035 | 0.234 | 2.2 |
| *Schizosaccharomyces* | 0.002 | 0.002 | 76.7 |  | na | na | na |
| *Pyricularia* | 0.002 | 0.002 | 79.1 |  | na | na | na |
| *Cyberlindnera* | 0.002 | 0.007 | 48.8 |  | na | na | na |
| *Zymoseptoria* | 0.002 | 0.002 | 72.1 |  | na | na | na |
| *Podospora* | 0.002 | 0.002 | 65.1 |  | 0.012 | 0.079 | 2.2 |
| *Pichia* | 0.002 | 0.004 | 58.1 |  | na | na | na |
| *Metarhizium* | 0.002 | 0.002 | 53.5 |  | na | na | na |
| *Cladophialophora* | 0.002 | 0.003 | 48.8 |  | na | na | na |
| *Epichloe* | 0.002 | 0.005 | 37.2 |  | na | na | na |
| *Bipolaris* | 0.002 | 0.002 | 67.4 |  | na | na | na |
| *Ascosphaera* | 0.002 | 0.005 | 32.6 |  | 19.306 | 23.113 | 71.7 |
| *Fonsecaea* | 0.002 | 0.002 | 51.2 |  | na | na | na |
| *Parastagonospora* | 0.001 | 0.002 | 58.1 |  | na | na | na |
| *Ascoidea* | 0.001 | 0.003 | 60.5 |  | na | na | na |
| *Capronia* | 0.001 | 0.002 | 55.8 |  | 0.006 | 0.029 | 4.3 |
| *Agaricus* | 0.001 | 0.002 | 53.5 |  | na | na | na |
| *Cercospora* | 0.001 | 0.002 | 62.8 |  | na | na | na |
| *Venturia* | 0.001 | 0.002 | 55.8 |  | na | na | na |
| *Komagataella* | 0.001 | 0.004 | 41.9 |  | na | na | na |
| *Lichtheimia* | 0.001 | 0.001 | 65.1 |  | na | na | na |
| *Cordyceps* | 0.001 | 0.002 | 41.9 |  | na | na | na |
| *Kwoniella* | 0.001 | 0.001 | 53.5 |  | na | na | na |
| *Coccidioides* | 0.001 | 0.001 | 62.8 |  | na | na | na |
| *Spathaspora* | 0.001 | 0.003 | 30.2 |  | na | na | na |
| *Verticillium* | 0.001 | 0.001 | 55.8 |  | na | na | na |
| *Pochonia* | 0.001 | 0.001 | 72.1 |  | na | na | na |
| *Acaromyces* | 0.001 | 0.001 | 72.1 |  | na | na | na |
| *Tilletiaria* | 0.001 | 0.003 | 27.9 |  | na | na | na |
| *Millerozyma* | 0.001 | 0.003 | 30.2 |  | na | na | na |
| *Ustilago* | 0.001 | 0.001 | 48.8 |  | na | na | na |
| *Scheffersomyces* | 0.001 | 0.002 | 37.2 |  | na | na | na |
| *Melampsora* | 0.001 | 0.001 | 48.8 |  | 0.012 | 0.061 | 4.3 |
| *Trametes* | 0.001 | 0.001 | 41.9 |  | na | na | na |
| *Arthrobotrys* | 0.001 | 0.001 | 48.8 |  | na | na | na |
| *Lodderomyces* | 0.001 | 0.002 | 34.9 |  | na | na | na |
| *Phycomyces* | 0.001 | 0.001 | 39.5 |  | na | na | na |
| *Glarea* | 0.001 | 0.002 | 32.6 |  | na | na | na |
| *Hyaloscypha* | 0.001 | 0.001 | 44.2 |  | na | na | na |
| *Moesziomyces* | 0.001 | 0.001 | 27.9 |  | na | na | na |
| *Paraphaeosphaeria* | 0.001 | 0.001 | 46.5 |  | na | na | na |
| *Suhomyces* | 0.001 | 0.001 | 32.6 |  | na | na | na |
| *Marssonina* | 0.001 | 0.001 | 41.9 |  | na | na | na |
| *Rhodotorula* | 0.001 | 0.001 | 32.6 |  | 0.021 | 0.069 | 10.9 |
| *Rhizopus* | 0.001 | 0.001 | 30.2 |  | 0.003 | 0.023 | 2.2 |
| *Meyerozyma* | 0.001 | 0.002 | 25.6 |  | na | na | na |
| *Trichophyton* | 0.000 | 0.001 | 34.9 |  | na | na | na |
| *Sporothrix* | 0.000 | 0.001 | 27.9 |  | na | na | na |
| *Thermothielavioides* | 0.000 | 0.001 | 30.2 |  | na | na | na |
| *Puccinia* | 0.000 | 0.001 | 34.9 |  | 0.015 | 0.072 | 6.5 |
| *Sugiyamaella* | 0.000 | 0.002 | 20.9 |  | na | na | na |
| *Yamadazyma* | 0.000 | 0.002 | 20.9 |  | na | na | na |
| *Xanthophyllomyces* | 0.000 | 0.001 | 34.9 |  | na | na | na |
| *Phialophora* | 0.000 | 0.001 | 25.6 |  | na | na | na |
| *Pestalotiopsis* | 0.000 | 0.001 | 25.6 |  | na | na | na |
| *Pneumocystis* | 0.000 | 0.001 | 25.6 |  | na | na | na |
| *Sphaerulina* | 0.000 | 0.001 | 32.6 |  | na | na | na |
| *Rhinocladiella* | 0.000 | 0.001 | 18.6 |  | na | na | na |
| *Thermothelomyces* | 0.000 | 0.001 | 30.2 |  | na | na | na |
| *Neurospora* | 0.000 | 0.001 | 25.6 |  | na | na | na |
| *Phialocephala* | 0.000 | 0.001 | 18.6 |  | na | na | na |
| *Wallemia* | 0.000 | 0.001 | 23.3 |  | 0.003 | 0.012 | 4.3 |
| *Blastomyces* | 0.000 | 0.001 | 20.9 |  | na | na | na |
