# Supplementary material for: Reconstructing the ecosystem context of a species: Honey-borne DNA reveals the roles of the honeybee
Source: PLoS One. 2022 Jul 13;17(7):e0268250. doi: 10.1371/journal.pone.0268250 (PMC9278776; doi:10.1371/journal.pone.0268250)
Supplement: S4 Table — The hundred most abundant plant genera based on their mean RRA from metagenomics, with their %FOO, compared with the mean RRA and %FOO from ITS2, rbcLa and trnL metabarcoding. (DOCX) [file pone.0268250.s008.docx]

**S4 Table. The most abundant plant genera from metagenomics and metabarcoding**

The hundred most abundant plant genera based on their mean RRA from metagenomics, with their %FOO, compared with the mean RRA and %FOO from ITS2, rbcLa and trnL metabarcoding.

|  | metagenomics | |  |  | ITS2 |  |  |  | rbcLa |  |  |  | trnL |  |  |
| --- | --- | --- | --- | --- | --- | --- | --- | --- | --- | --- | --- | --- | --- | --- | --- |
|  | RRA |  | %FOO |  | RRA |  | %FOO |  | RRA |  | %FOO |  | RRA |  | %FOO |
| genus | mean | SD |  |  | mean | SD |  |  | mean | SD |  |  | mean | SD |  |
| *Brassica* | 4.810 | 5.437 | 100.0 |  | 27.001 | 22.691 | 89.1 |  | 23.274 | 18.950 | 89.1 |  | na | na | na |
| *Malus* | 0.419 | 0.680 | 97.7 |  | 0.093 | 0.324 | 15.2 |  | na | na | na |  | na | na | na |
| *Trifolium* | 0.392 | 0.471 | 100.0 |  | 21.532 | 18.032 | 87.0 |  | 9.674 | 12.361 | 91.3 |  | 2.257 | 2.556 | 76.1 |
| *Medicago* | 0.292 | 0.339 | 97.7 |  | 0.005 | 0.035 | 2.2 |  | 0.058 | 0.178 | 21.7 |  | na | na | na |
| *Prunus* | 0.225 | 0.418 | 97.7 |  | 0.692 | 2.566 | 39.1 |  | 5.138 | 11.730 | 76.1 |  | 6.768 | 15.084 | 78.3 |
| *Pyrus* | 0.182 | 0.255 | 95.3 |  | 0.012 | 0.060 | 4.3 |  | na | na | na |  | na | na | na |
| *Fragaria* | 0.148 | 0.303 | 88.4 |  | 0.026 | 0.101 | 6.5 |  | 0.004 | 0.020 | 4.3 |  | na | na | na |
| *Cicer* | 0.134 | 0.127 | 97.7 |  | na | na | na |  | na | na | na |  | na | na | na |
| *Populus* | 0.133 | 0.189 | 97.7 |  | 0.023 | 0.155 | 2.2 |  | 0.031 | 0.079 | 19.6 |  | 0.049 | 0.207 | 6.5 |
| *Rosa* | 0.116 | 0.187 | 95.3 |  | 0.047 | 0.141 | 21.7 |  | 0.019 | 0.040 | 21.7 |  | na | na | na |
| *Gossypium* | 0.097 | 0.075 | 100.0 |  | na | na | na |  | na | na | na |  | na | na | na |
| *Solanum* | 0.095 | 0.064 | 97.7 |  | 0.007 | 0.047 | 2.2 |  | 0.013 | 0.077 | 4.3 |  | 0.010 | 0.065 | 2.2 |
| *Vigna* | 0.092 | 0.048 | 100.0 |  | na | na | na |  | na | na | na |  | na | na | na |
| *Lupinus* | 0.077 | 0.040 | 100.0 |  | 0.014 | 0.050 | 8.7 |  | na | na | na |  | 0.147 | 0.272 | 30.4 |
| *Pinus* | 0.069 | 0.139 | 93.0 |  | na | na | na |  | 1.327 | 1.708 | 89.1 |  | 0.983 | 1.389 | 65.2 |
| *Vicia* | 0.064 | 0.095 | 95.3 |  | 1.989 | 7.969 | 45.7 |  | 1.023 | 1.854 | 73.9 |  | 3.363 | 5.647 | 69.6 |
| *Lotus* | 0.047 | 0.105 | 95.3 |  | 0.013 | 0.071 | 4.3 |  | 0.005 | 0.023 | 4.3 |  | 0.005 | 0.032 | 2.2 |
| *Vitis* | 0.042 | 0.053 | 97.7 |  | na | na | na |  | 0.233 | 0.923 | 13.0 |  | na | na | na |
| *Ipomoea* | 0.041 | 0.035 | 95.3 |  | na | na | na |  | na | na | na |  | na | na | na |
| *Rubus* | 0.033 | 0.071 | 83.7 |  | 6.500 | 11.230 | 76.1 |  | 4.218 | 6.759 | 93.5 |  | na | na | na |
| *Frangula* | 0.031 | 0.176 | 18.6 |  | 0.858 | 4.705 | 10.9 |  | na | na | na |  | na | na | na |
| *Gossypioides* | 0.030 | 0.027 | 95.3 |  | na | na | na |  | na | na | na |  | na | na | na |
| *Salix* | 0.030 | 0.049 | 93.0 |  | 0.254 | 0.535 | 34.8 |  | 1.420 | 1.888 | 93.5 |  | 4.317 | 4.762 | 97.8 |
| *Ananas* | 0.029 | 0.018 | 100.0 |  | na | na | na |  | na | na | na |  | na | na | na |
| *Glycine* | 0.028 | 0.023 | 97.7 |  | 0.013 | 0.054 | 6.5 |  | na | na | na |  | na | na | na |
| *Cucumis* | 0.025 | 0.021 | 97.7 |  | 0.022 | 0.117 | 6.5 |  | na | na | na |  | 0.015 | 0.068 | 4.3 |
| *Arachis* | 0.025 | 0.020 | 95.3 |  | na | na | na |  | na | na | na |  | na | na | na |
| *Raphanus* | 0.023 | 0.032 | 95.3 |  | 1.587 | 6.749 | 32.6 |  | na | na | na |  | na | na | na |
| *Arabidopsis* | 0.023 | 0.017 | 97.7 |  | na | na | na |  | na | na | na |  | na | na | na |
| *Theobroma* | 0.022 | 0.020 | 95.3 |  | na | na | na |  | na | na | na |  | na | na | na |
| *Oryza* | 0.022 | 0.016 | 95.3 |  | na | na | na |  | na | na | na |  | na | na | na |
| *Daucus* | 0.021 | 0.038 | 95.3 |  | na | na | na |  | 0.018 | 0.045 | 17.4 |  | na | na | na |
| *Quercus* | 0.021 | 0.028 | 95.3 |  | 0.274 | 0.695 | 32.6 |  | 2.973 | 10.790 | 82.6 |  | 5.839 | 9.351 | 80.4 |
| *Ziziphus* | 0.021 | 0.090 | 90.7 |  | na | na | na |  | na | na | na |  | na | na | na |
| *Linum* | 0.020 | 0.012 | 97.7 |  | na | na | na |  | na | na | na |  | na | na | na |
| *Papaver* | 0.019 | 0.024 | 95.3 |  | 0.001 | 0.009 | 2.2 |  | 0.049 | 0.083 | 34.8 |  | na | na | na |
| *Arabis* | 0.019 | 0.018 | 95.3 |  | na | na | na |  | na | na | na |  | na | na | na |
| *Dioscorea* | 0.018 | 0.027 | 72.1 |  | na | na | na |  | na | na | na |  | na | na | na |
| *Pisum* | 0.014 | 0.015 | 90.7 |  | 0.003 | 0.021 | 2.2 |  | 0.040 | 0.253 | 4.3 |  | 0.017 | 0.088 | 4.3 |
| *Lactuca* | 0.013 | 0.013 | 95.3 |  | na | na | na |  | na | na | na |  | na | na | na |
| *Camellia* | 0.011 | 0.010 | 95.3 |  | na | na | na |  | na | na | na |  | na | na | na |
| *Olea* | 0.011 | 0.018 | 93.0 |  | na | na | na |  | 0.304 | 0.689 | 50.0 |  | na | na | na |
| *Sinapis* | 0.010 | 0.029 | 67.4 |  | 0.222 | 1.227 | 10.9 |  | na | na | na |  | na | na | na |
| *Nicotiana* | 0.010 | 0.008 | 93.0 |  | na | na | na |  | na | na | na |  | na | na | na |
| *Triticum* | 0.009 | 0.011 | 93.0 |  | na | na | na |  | na | na | na |  | na | na | na |
| *Cannabis* | 0.008 | 0.015 | 86.0 |  | 0.026 | 0.176 | 2.2 |  | 0.129 | 0.829 | 6.5 |  | na | na | na |
| *Helianthus* | 0.008 | 0.006 | 95.3 |  | na | na | na |  | na | na | na |  | na | na | na |
| *Capsella* | 0.007 | 0.005 | 97.7 |  | 0.010 | 0.051 | 4.3 |  | na | na | na |  | na | na | na |
| *Cajanus* | 0.007 | 0.005 | 90.7 |  | na | na | na |  | na | na | na |  | na | na | na |
| *Abrus* | 0.007 | 0.006 | 90.7 |  | na | na | na |  | na | na | na |  | na | na | na |
| *Taraxacum* | 0.007 | 0.010 | 76.7 |  | 0.105 | 0.261 | 28.3 |  | 5.896 | 7.108 | 93.5 |  | na | na | na |
| *Chenopodium* | 0.006 | 0.009 | 88.4 |  | 0.007 | 0.048 | 2.2 |  | 0.015 | 0.064 | 6.5 |  | na | na | na |
| *Vaccinium* | 0.006 | 0.009 | 67.4 |  | 1.489 | 4.612 | 34.8 |  | 0.075 | 0.163 | 32.6 |  | 0.874 | 2.265 | 39.1 |
| *Spirodela* | 0.006 | 0.005 | 100.0 |  | na | na | na |  | na | na | na |  | na | na | na |
| *Picea* | 0.006 | 0.009 | 83.7 |  | na | na | na |  | 0.331 | 0.945 | 50.0 |  | na | na | na |
| *Zea* | 0.006 | 0.006 | 95.3 |  | na | na | na |  | 0.003 | 0.015 | 4.3 |  | na | na | na |
| *Brachypodium* | 0.005 | 0.006 | 95.3 |  | na | na | na |  | na | na | na |  | na | na | na |
| *Carica* | 0.005 | 0.005 | 95.3 |  | na | na | na |  | na | na | na |  | na | na | na |
| *Comarum* | 0.005 | 0.016 | 14.0 |  | 4.816 | 13.794 | 19.6 |  | 2.946 | 8.288 | 67.4 |  | na | na | na |
| *Coffea* | 0.005 | 0.005 | 90.7 |  | na | na | na |  | na | na | na |  | na | na | na |
| *Juglans* | 0.005 | 0.007 | 90.7 |  | na | na | na |  | na | na | na |  | na | na | na |
| *Betula* | 0.005 | 0.008 | 69.8 |  | 0.558 | 1.702 | 39.1 |  | na | na | na |  | na | na | na |
| *Hevea* | 0.005 | 0.005 | 88.4 |  | na | na | na |  | na | na | na |  | na | na | na |
| *Morus* | 0.005 | 0.011 | 83.7 |  | na | na | na |  | na | na | na |  | na | na | na |
| *Camelina* | 0.005 | 0.004 | 90.7 |  | na | na | na |  | na | na | na |  | na | na | na |
| *Prosopis* | 0.005 | 0.007 | 83.7 |  | na | na | na |  | na | na | na |  | na | na | na |
| *Cynara* | 0.005 | 0.003 | 90.7 |  | na | na | na |  | na | na | na |  | na | na | na |
| *Durio* | 0.004 | 0.004 | 88.4 |  | na | na | na |  | na | na | na |  | na | na | na |
| *Hordeum* | 0.004 | 0.006 | 81.4 |  | na | na | na |  | 0.008 | 0.031 | 6.5 |  | na | na | na |
| *Eriosyce* | 0.004 | 0.004 | 67.4 |  | na | na | na |  | na | na | na |  | na | na | na |
| *Cucurbita* | 0.004 | 0.004 | 90.7 |  | na | na | na |  | na | na | na |  | na | na | na |
| *Phaseolus* | 0.004 | 0.003 | 90.7 |  | na | na | na |  | na | na | na |  | na | na | na |
| *Lolium* | 0.004 | 0.008 | 55.8 |  | na | na | na |  | na | na | na |  | na | na | na |
| *Citrus* | 0.004 | 0.008 | 37.2 |  | na | na | na |  | na | na | na |  | na | na | na |
| *Spinacia* | 0.004 | 0.003 | 93.0 |  | na | na | na |  | na | na | na |  | na | na | na |
| *Phoenix* | 0.004 | 0.004 | 74.4 |  | na | na | na |  | na | na | na |  | na | na | na |
| *Capsicum* | 0.003 | 0.003 | 81.4 |  | na | na | na |  | na | na | na |  | na | na | na |
| *Jatropha* | 0.003 | 0.004 | 86.0 |  | na | na | na |  | na | na | na |  | na | na | na |
| *Beta* | 0.003 | 0.003 | 86.0 |  | na | na | na |  | na | na | na |  | na | na | na |
| *Asparagus* | 0.003 | 0.005 | 74.4 |  | na | na | na |  | 0.002 | 0.016 | 2.2 |  | na | na | na |
| *Amborella* | 0.003 | 0.004 | 76.7 |  | na | na | na |  | na | na | na |  | na | na | na |
| *Manihot* | 0.003 | 0.004 | 74.4 |  | na | na | na |  | na | na | na |  | na | na | na |
| *Ricinus* | 0.003 | 0.004 | 69.8 |  | na | na | na |  | na | na | na |  | na | na | na |
| *Nelumbo* | 0.003 | 0.004 | 81.4 |  | na | na | na |  | na | na | na |  | na | na | na |
| *Melilotus* | 0.003 | 0.011 | 30.2 |  | 0.689 | 2.699 | 28.3 |  | 0.957 | 2.523 | 63.0 |  | na | na | na |
| *Syzygium* | 0.003 | 0.003 | 83.7 |  | na | na | na |  | na | na | na |  | na | na | na |
| *Eutrema* | 0.003 | 0.003 | 86.0 |  | na | na | na |  | na | na | na |  | na | na | na |
| *Sesamum* | 0.003 | 0.003 | 81.4 |  | na | na | na |  | na | na | na |  | na | na | na |
| *Aegilops* | 0.003 | 0.003 | 74.4 |  | na | na | na |  | na | na | na |  | na | na | na |
| *Silene* | 0.003 | 0.009 | 53.5 |  | na | na | na |  | 0.014 | 0.076 | 4.3 |  | na | na | na |
| *Lens* | 0.003 | 0.004 | 65.1 |  | na | na | na |  | na | na | na |  | na | na | na |
| *Erythranthe* | 0.003 | 0.004 | 72.1 |  | na | na | na |  | na | na | na |  | na | na | na |
| *Elaeis* | 0.002 | 0.002 | 88.4 |  | na | na | na |  | na | na | na |  | na | na | na |
| *Eucalyptus* | 0.002 | 0.003 | 72.1 |  | na | na | na |  | na | na | na |  | na | na | na |
| *Lathyrus* | 0.002 | 0.004 | 60.5 |  | 0.006 | 0.028 | 4.3 |  | 0.056 | 0.193 | 13.0 |  | 0.062 | 0.351 | 6.5 |
| *Musa* | 0.002 | 0.002 | 67.4 |  | 0.944 | 4.852 | 4.3 |  | 0.282 | 1.383 | 6.5 |  | na | na | na |
| *Rhodamnia* | 0.002 | 0.003 | 74.4 |  | na | na | na |  | na | na | na |  | na | na | na |
| *Tarenaya* | 0.002 | 0.002 | 74.4 |  | na | na | na |  | na | na | na |  | na | na | na |
| *Saccharum* | 0.002 | 0.002 | 65.1 |  | na | na | na |  | na | na | na |  | na | na | na |
| *Orobanche* | 0.002 | 0.002 | 72.1 |  | na | na | na |  | na | na | na |  | na | na | na |
