# Supplementary material for: Reconstructing the ecosystem context of a species: Honey-borne DNA reveals the roles of the honeybee
Source: PLoS One. 2022 Jul 13;17(7):e0268250. doi: 10.1371/journal.pone.0268250 (PMC9278776; doi:10.1371/journal.pone.0268250)
Supplement: S5 Table — Functional classes of 16S bacterial OTU with the mean RRA (±SD) and FOO%. The functionality classes are ordered in the table based on the mean RRA from the primers 16Sa, following those from 16Sb. Shown are classes presented by >0.01% of mean RRA. (DOCX) [file pone.0268250.s009.docx]

**S5 Table. Functional classes of the bacterial OTUs from 16S metabarcoding**

Functional classes of 16S bacterial OTU with the mean RRA (±SD) and FOO%. The functionality classes are ordered in the table based on the mean RRA from the primers 16Sa, following those from 16Sb. Shown are classes presented by >0.01 % of mean RRA.

|  | 16Sa |  |  | 16Sb |  |  |
| --- | --- | --- | --- | --- | --- | --- |
|  | RRA |  | FOO% | RRA |  | FOO% |
| Functionality | mean | SD |  | mean | SD |  |
| fermentation | 64.76 | 32.28 | 86.96 | 37.15 | 33.56 | 8.70 |
| animal parasites or symbionts | 9.47 | 9.06 | 84.78 | 4.03 | 7.05 | 2.17 |
| aromatic compound degradation | 6.87 | 7.88 | 73.91 | 2.35 | 4.84 | 2.17 |
| nitrogen fixation | 3.10 | 6.53 | 43.48 | 11.33 | 17.77 | 63.04 |
| human pathogens all | 3.04 | 6.08 | 34.78 | 2.55 | 4.98 | 13.04 |
| nitrate denitrification | 2.61 | 8.10 | 28.26 | 0.63 | 3.93 | 63.04 |
| intracellular parasites | 2.09 | 7.90 | 8.70 | 25.34 | 26.65 | 15.22 |
| aromatic hydrocarbon degradation | 1.39 | 4.09 | 21.74 | 0.23 | 0.75 | 2.17 |
| dark hydrogen oxidation | 1.37 | 6.80 | 17.39 | 1.42 | 5.69 | 2.17 |
| dark thiosulfate oxidation | 1.34 | 4.07 | 23.91 | 0.01 | 0.08 | 8.70 |
| nitrate respiration | 1.32 | 4.34 | 17.39 | 0.36 | 1.23 | 60.87 |
| aerobic chemoheterotrophy | 0.70 | 2.75 | 6.52 | 7.68 | 20.48 | 2.17 |
| photoheterotrophy | 0.22 | 1.23 | 26.09 | 0.10 | 0.57 | 26.09 |
| xylanolysis | 0.15 | 0.63 | 10.87 | 1.13 | 5.69 | 71.74 |
| aliphatic non methane hydrocarbon degradation | 0.06 | 0.34 | 4.35 | 0.23 | 0.75 | 2.17 |
| predatory or exoparasitic | 0.06 | 0.32 | 8.70 | 0.09 | 0.46 | 71.74 |
| cellulolysis | 0.05 | 0.24 | 4.35 | 0.02 | 0.16 | 4.35 |
| manganese oxidation | na | na | na | 2.61 | 5.96 | 6.52 |
| plant pathogen | na | na | na | 0.98 | 1.93 | 60.87 |
| nitrate reduction | na | na | na | 0.91 | 4.96 | 41.30 |
| human gut | na | na | na | 0.61 | 4.07 | 8.70 |
| thiosulfate respiration | na | na | na | 0.19 | 1.31 | 93.48 |
| hydrogenotrophic methanogenesis | na | na | na | 0.02 | 0.12 | 17.39 |
| methanogenesis by CO2 reduction with H2 | na | na | na | 0.02 | 0.12 | 15.22 |
