# Supplementary material for: Reconstructing the ecosystem context of a species: Honey-borne DNA reveals the roles of the honeybee
Source: PLoS One. 2022 Jul 13;17(7):e0268250. doi: 10.1371/journal.pone.0268250 (PMC9278776; doi:10.1371/journal.pone.0268250)
Supplement: S6 Table — Functional classes to which the metabarcoding ITS2 fungal reads were assigned to, with both the mean RRA (±SD) and FOO% of the functionalities. The functionality classes are ordered based on the mean RRA. (DOCX) [file pone.0268250.s010.docx]

**S6 Table. Functional classes of the fungal genera from ITS2 metabarcoding**

Functional classes to which the metabarcoding ITS2 fungal reads were assigned to, with both the mean RRA (±SD) and FOO% of the functionalities. The functionality classes are ordered based on the mean RRA.

|  | ITS2 |  |  |
| --- | --- | --- | --- |
| Functionality | RRA |  | FOO% |
| plant pathogen | 44.66 | 27.11 | 100.00 |
| animal pathogen | 21.47 | 25.51 | 71.74 |
| animal pathogen/ endosymbiont/ undefined saprotroph | 9.36 | 16.54 | 73.91 |
| undefined saprotroph | 9.02 | 9.40 | 95.65 |
| animal endosymbiont/ animal pathogen/ endophyte/ plant pathogen/ undefined saprotroph | 8.60 | 12.64 | 82.61 |
| ectomycorrhizal | 3.43 | 5.05 | 89.13 |
| ericoid mycorrhizal | 1.27 | 1.63 | 71.74 |
| dung saprotroph/ undefined saprotroph/ wood saprotroph | 1.25 | 4.78 | 56.52 |
| animal pathogen/ endophyte/ plant saprotroph/ soil saprotroph/ undefined saprotroph/ wood saprotroph | 0.42 | 1.36 | 36.96 |
| endophyte | 0.16 | 0.66 | 19.57 |
| lichenized | 0.10 | 0.50 | 21.74 |
| wood saprotroph | 0.06 | 0.14 | 23.91 |
| endophyte/ litter saprotroph/ soil saprotroph/ undefined saprotroph | 0.04 | 0.10 | 21.74 |
| arbuscular mycorrhizal | 0.03 | 0.12 | 10.87 |
| dung saprotroph/ wood saprotroph | 0.02 | 0.15 | 2.17 |
| animal pathogen/ undefined saprotroph | 0.01 | 0.07 | 4.35 |
| dung saprotroph/ undefined saprotroph | 0.01 | 0.05 | 8.70 |
| animal pathogen/ endophyte | 0.01 | 0.05 | 8.70 |
| fungal parasite/ litter saprotroph | 0.01 | 0.04 | 4.35 |
| plant pathogen/ wood saprotroph | 0.01 | 0.05 | 2.17 |
| fungal parasite/ lichen parasite | 0.01 | 0.05 | 2.17 |
| epiphyte | 0.01 | 0.03 | 4.35 |
| endophyte/ undefined saprotroph | 0.01 | 0.04 | 2.17 |
| endophyte/ plant pathogen/ wood saprotroph | 0.01 | 0.03 | 4.35 |
